# The molecular basis of the inhibition of Ca_V_1 calcium-dependent inactivation by the distal carboxy tail

**DOI:** 10.1016/j.jbc.2021.100502

**Published:** 2021-03-02

**Authors:** Lingjie Sang, Daiana C.O. Vieira, David T. Yue, Manu Ben-Johny, Ivy E. Dick

**Affiliations:** 1 Department of Biomedical Engineering, The Johns Hopkins University School of Medicine, Baltimore, Maryland, USA; 2 Department of Physiology, University of Maryland School of Medicine, Baltimore, Maryland, USA; 3 Department of Physiology and Cellular Biophysics, Columbia University, New York, New York, USA

**Keywords:** calcium channel, calmodulin (CaM), ion channel, calcium-dependent inactivation (CDI), fluorescence resonance energy transfer (FRET), patch clamp, apoCaM, Ca^2+^ free calmodulin, Ca^2+^, calcium, CaM, calmodulin, Ca_V_, voltage-gated calcium channel, CDI, calcium/CaM-dependent inactivation, CFP, cyan fluorescent protein, DCRD, distal C-terminal regulatory domain, DCT, distal carboxy tail, FR, fret ratio, FRET, fluorescence resonance energy transfer, ICDI, inhibitor of CDI, iTL, individually transformed Langmuir, PCRD, proximal C-terminal regulatory domain, YFP, yellow fluorescent protein

## Abstract

Ca^2+^/calmodulin-dependent inactivation (CDI) of Ca_V_ channels is a critical regulatory process that tunes the kinetics of Ca^2+^ entry for different cell types and physiologic responses. CDI is mediated by calmodulin (CaM), which is bound to the IQ domain of the Ca_V_ carboxy tail. This modulatory process is tailored by alternative splicing such that select splice variants of Ca_V_1.3 and Ca_V_1.4 contain a long distal carboxy tail (DCT). The DCT harbors an inhibitor of CDI (ICDI) module that competitively displaces CaM from the IQ domain, thereby diminishing CDI. While this overall mechanism is now well described, the detailed interactions required for ICDI binding to the IQ domain are yet to be elucidated. Here, we perform alanine-scanning mutagenesis of the IQ and ICDI domains and evaluate the contribution of neighboring regions to CDI inhibition. Through FRET binding analysis, we identify functionally relevant residues within the Ca_V_1.3 IQ domain and the Ca_V_1.4 ICDI and nearby A region, which are required for high-affinity IQ/ICDI binding. Importantly, patch-clamp recordings demonstrate that disruption of this interaction commensurately diminishes ICDI function resulting in the re-emergence of CDI in mutant channels. Furthermore, Ca_V_1.2 channels harbor a homologous DCT; however, the ICDI region of this channel does not appear to appreciably modulate Ca_V_1.2 CDI. Yet coexpression of Ca_V_1.2 ICDI with select Ca_V_1.3 splice variants significantly disrupts CDI, implicating a cross-channel modulatory scheme in cells expressing both channel subtypes. In all, these findings provide new insights into a molecular rheostat that fine-tunes Ca^2+^-entry and supports normal neuronal and cardiac function.

L-type voltage-gated calcium channels (Ca_V_1.1–1.4) are an important conduit for extracellular Ca^2+^ entry into many excitable cells including cardiac myocytes, neurons, smooth muscle, and skeletal muscle (1, 2, 3, 4). As such, these channels are subject to rich and powerful modes of feedback regulation (5, 6, 7). In particular, Ca^2+^/calmodulin-dependent inactivation (CDI) of L-type channels is a crucial negative feedback mechanism that reshapes the electrical properties of neurons and cardiac myocytes and protects cells from Ca^2+^ overload (8, 9, 10). CDI is driven by the ubiquitous Ca^2+^ sensing molecule, calmodulin (CaM) (10, 11). Under basal Ca^2+^ conditions, Ca^2+^-free CaM (apoCaM) binds to the carboxy-terminal IQ domain of the channel and enhances channel openings (12). Upon elevation of intracellular Ca^2+^, the “resident” CaM repositions on the channel, interacting with Ca^2+^/CaM binding sites located on the channel amino- and proximal carboxy- termini (13, 14). This conformational change antagonizes the initial upregulation in channel open probability, which manifests as CDI. Not surprisingly, CDI of L-type channels has emerged as a key physiological process to limit excess Ca^2+^ influx during repetitive or sustained depolarization, and disruption of this feedback in the cardiac myocytes may lead to lethal cardiac arrhythmias (15, 16). This stereotypic behavior, however, diverges in multiple physiological settings where strong CDI of L-type channels is curtailed, thus permitting Ca^2+^ channels to faithfully respond to a tonic stimulus. For example, in photoreceptors and bipolar cells, endogenous Ca_V_1.4 exhibits minimal CDI, thereby allowing sustained Ca^2+^ influx and slow, graded changes in the membrane potential necessary for tonic glutamate release, and normal vision (17, 18). Similarly, Ca_V_1.3 channels in inner hair cells also lack CDI (19). Beyond these, the basal strength of Ca_V_1.2 and Ca_V_1.3 CDI varies in different neuronal subtypes in the central nervous system, suggesting a sophisticated scheme of Ca_V_ channel feedback ripe with physiological insights (20).

The molecular mechanisms that fine-tune L-type channel CDI are twofold and have been of long-standing interest. One scheme involves channel-interacting proteins such as calmodulin-like Ca^2+^-binding proteins (CaBP1-4) (18, 19, 21, 22) and SH3 and cysteine-rich domain containing proteins (stac1-3) (23, 24, 25, 26) that suppress CDI utilizing an allosteric or mixed-allosteric mechanism. In contrast, Ca_V_1.3 and Ca_V_1.4 channels may intrinsically disable CDI *via* an alternatively spliced specialized CDI-inhibiting module (ICDI) within the distal carboxy tail (DCT) of the channel (20, 27, 28, 29, 30, 31, 32, 33). The latter form of regulation is complex and bears important biological consequences. First, splice inclusion of the DCT occurs in a cell-type-dependent manner. For instance, alternative splicing of Ca_V_1.3 results in variable inclusion of the ICDI domain in distinct regions of the brain and in the sinoatrial node, enabling precise tuning of CDI in these cell types (27, 31, 34, 35). Second, Ca_V_1.2 appears to harbor a highly homologous ICDI region (33), yet CDI for this channel is known to be robust, both when evaluated as full-length channels in heterologous expression system and in primary cells where the carboxy-tail containing ICDI is believed to be cleaved off the channel (9, 10, 11, 33). As such, the function of the ICDI module within Ca_V_1.2 channels remains unclear. Third, the inhibition of CDI by ICDI is the result of competitive binding by apoCaM *versus* ICDI with the channel IQ domain (20, 31). In addition to diminishing CDI, the displacement of apoCaM results in a dramatic decrease in baseline channel open probability (12). Fourth, adding to the richness the modulatory role of ICDI, RNA editing, and/or fluctuations in cytosolic CaM concentrations can tune the extent of this competition, enabling different degrees of CDI tailored to specific cell types or physiologic states (12, 36). Importantly, pathologic changes to this system may be linked to altered CaM concentrations in Parkinson’s disease and heart failure (37, 38), and mutations within the ICDI of Ca_V_1.4 channels are known to be causative of congenital stationary night blindness (32, 39, 40). Thus, the modulation of CDI by ICDI stands as a critical and robust mechanism for adapting channel regulation to select cell types and conditions. Moreover, as the number of known pathogenic mutations within LTCCs continues to grow, the ability to map these mutations to a locus with known mechanistic impact would enable rapid insight into the pathogenesis of LTCC channelopathies.

Although the overall competitive nature of ICDI regulation of L-type channels is now well established (20, 31), the precise binding interfaces involved in this regulation are yet to be identified. This gap in understanding is critical as mutations in the carboxy tail of LTCCs result in neurological disease (32, 39, 40). Furthermore, a residue-level analysis may shed light upon structural differences between the ICDI domain of Ca_V_1.3 and the homologous segment of Ca_V_1.2 that engender differential functional regulation. To characterize the landscape of the IQ/ICDI interaction of L-type channels, here we undertook systematic alanine scanning mutagenesis of both IQ and ICDI domains. Through live-cell FRET two-hybrid binding assays and electrophysiological analysis, we identified several novel hotspots on both IQ and ICDI segments that mediate a high-affinity interaction and are functionally relevant for CDI inhibition. Systematic analysis of these mutations revealed a strong inverse correlation between the strength of CDI and the binding affinity of the ICDI domain for the IQ segment, as predicted for a competitive inhibitor (13, 14, 20). Thus, we have identified residues that alter binding in a functionally relevant manner. Moreover, similar critical residues were identified in adjacent regions, defining a comprehensive interface map of the IQ/ICDI interaction. Finally, extending our analysis to Ca_V_1.2 channels, we found that the ICDI module binds to the Ca_V_1.2 IQ domain with a reduced affinity and that this binding is insufficient to cause more than a nominal change in the CDI of full-length channels. However, the ICDI from Ca_V_1.2 is capable of binding the Ca_V_1.3 IQ region with high affinity, resulting in a much larger decrease in CDI of these channels. Given the propensity of the carboxy tail of Ca_V_1.2 to exist as a separate peptide within myocytes and neurons (27, 33, 41, 42), these findings raise the prospect of a cross-channel feedback scheme in some cell types. Overall, these results elucidate the detailed binding interface between components of the carboxy tail of L-type Ca^2+^ channels, lending new insight into normal and pathologic channel regulation.

## Results

### Identification of critical residues within the IQ domain necessary for ICDI binding

**Figure 1:**
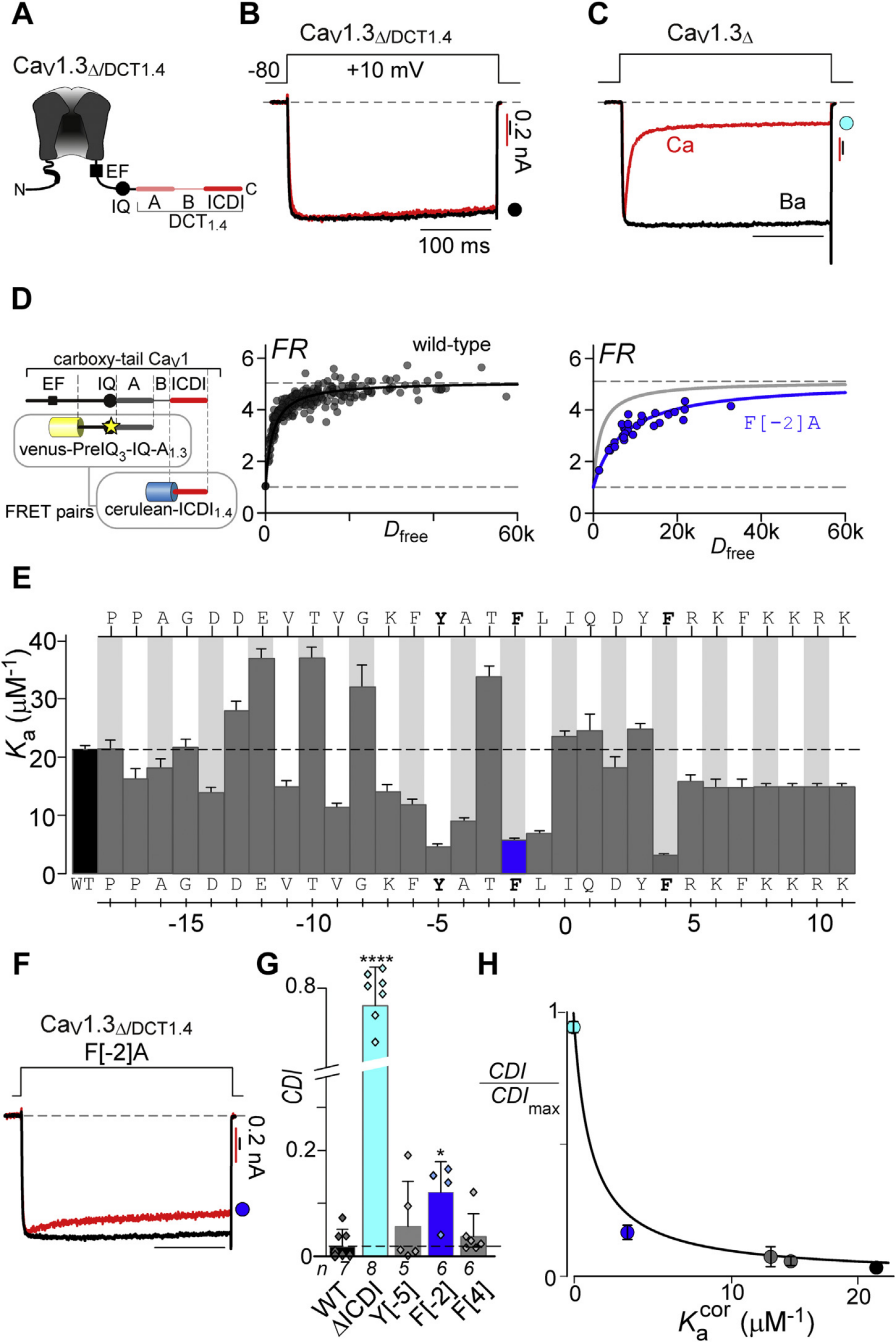
**Identifying the hotspots on the IQ region required for ICDI binding and function.***A*, cartoon depicting the chimeric Ca_V_1.3_Δ/DCT1.4_ channel. *B*, exemplar whole-cell recording showing minimal inactivation in response to a 10 mV step depolarization in Ba^2+^ (*black*) or Ca^2+^ (*red*) for Ca_V_1.3_Δ/DCT1.4_ demonstrating the function of ICDI_1.4_. Ba^2+^ current is scaled to enable comparison of the kinetics of the two traces. *Red scale bar* refers to the Ca^2+^ trace and *black* corresponds to the Ba^2+^ trace here and throughout. *C*, exemplar whole-cell recording of the truncated Ca_V_1.3_Δ_ channel, lacking the DCT containing ICDI. Robust CDI is seen as the strong decay of the Ca^2+^ current (*red*) as compared to the Ba^2+^ current. *D*, FRET two-hybrid assay of IQ/ICDI interaction. FRET binding partners are displayed on the *left*, with *star* indicating the locus of alanine mutations. Strong binding was measured for wild-type preIQ_3_-IQ-A_1.3_ with ICDI_1.4_ (*black*), while the mutation F[−2]A (*blue*) decreased the binding affinity compared with WT (*gray*). *E*, summary of *K*_a_ values for mutant Venus-preIQ_3_-IQ-A_1.3_*versus* Cerulean-ICDI_1.4_ peptides measured with FRET two-hybrid assay as in panel *D*. Alanine was systematically substituted into the IQ region of the Venus-preIQ_3_-IQ-A_1.3_ peptide, with the identity of the amino acid displayed on the *top* and *bottom of the bar graph* such that the canonical “I” of the IQ region is given position 0. The *dashed line* indicates the WT value and the *blue bar* corresponds to F[−2]A displayed in panel *D*. Data are displayed as mean ± SE. *F*, exemplar patch clamp data corresponding to F[−2]A in Ca_V_1.3_Δ/DCT1.4_ demonstrating a partial recovery of CDI due to the mutation. *G*, average CDI values for each mutation, colors correspond to the data in other panels (ANOVA summary analysis indicates a likelihood of significant difference at a level of *p* < 0.0001. Post-hoc Dunnett’s multiple comparisons *versus* WT indicated as ∗∗∗∗*p* < 0.0001; ∗*p* < 0.05). Data is displayed as mean ± SD. *H*, CDI and binding data for the mutations is well fit by Equation 2, validating the competitive mechanism. *Colored circles* correspond to the colored data in the figure panels.

To identify key residues that support a high-affinity IQ/ICDI interaction, we undertook systematic alanine substitution of the IQ domain and evaluated both the relative binding affinity and the strength of ICDI-mediated inhibition of CaM regulation. Importantly, the ICDI domains of both Ca_V_1.3 and Ca_V_1.4 are highly homologous and have been shown to interact with IQ domains in a similar manner evoking similar functional effects (20, 27, 29). Even so, the ICDI domain from Ca_V_1.4 (ICDI_1.4_) has a greater binding affinity for the IQ domains of both Ca_V_1.3 and Ca_V_1.4, with FRET binding assays yielding more robust measurements with enhanced signal-to-noise ratio as compared with ICDI_1.3_ (43). We therefore focus on this canonical ICDI motif for our studies. However, robust expression of the holo-Ca_V_1.4 channel in recombinant systems is notoriously challenging, largely due to their diminutive open probability (12, 44). We therefore chose to explore the interaction between the IQ domain of Ca_V_1.3 channels (IQ_1.3_) and ICDI_1.4_. To this end, we utilized a chimeric channel in which the DCT of Ca_V_1.4 is spliced onto the backbone of Ca_V_1.3 (Ca_V_1.3_Δ/DCT1.4_) (Fig. 1*A*), which has previously proven useful in dissecting the mechanisms underlying ICDI modulation of the channel (12, 20). This chimera furnishes a strong IQ/ICDI interaction coupled with a robust functional readout, enabling quantitative analysis.

To begin, we confirm the functional impact of ICDI in our chimeric channel by evaluating the extent of CDI in HEK293 cells. Indeed, CDI is entirely abolished in Ca_V_1.3_Δ/DCT1.4_, as seen by the identical Ba^2+^ and Ca^2+^ current decay in response to a depolarizing pulse (Fig. 1*B*). However, removal of DCT_1.4_ restores robust CDI, as seen by the rapid decay of the Ca^2+^ current (Fig. 1*C*, red). In contrast, when Ba^2+^ (which binds poorly to CaM) is used as the charge carrier, there is minimal inactivation (Fig. 1*C*, black). We therefore define the extent of CDI as the ratio of Ca^2+^ current remaining after 300 ms of depolarization *versus* Ba^2+^ current at the same time point.

We next utilized a FRET 2-hybrid binding assay (45, 46) to evaluate the relative strength of interactions between the IQ and ICDI regions. FRET binding pairs were constructed by tagging Cerulean fluorescent protein to ICDI_1.4_, and Venus fluorescent protein to PreIQ_3_-IQ-A_1.3_, a peptide that includes IQ_1.3_ as well as ∼30 residues upstream (PreIQ_3_) and ∼150 residues downstream (A-region) of the IQ domain (Fig. 1*D*, Fig. S1). Both PreIQ_3_ and A regions were included initially to ensure that all likely interacting residues were included. Strong binding was detected between the Venus-PreIQ_3_-IQ-A_1.3_ and Cerulean-ICDI_1.4_, as can be appreciated by the steep FRET binding curve determined by the FRET Ratio (FR) of each cell plotted as a function of the free donor concentration (Cerulean tagged ICDI_1.4_) (Fig. 1*D*, *black*). After calibration, the FRET binding curve for WT Venus-PreIQ_3_-IQ-A_1.3_ *versus* Cerulean-ICDI_1.4_ yielded a *K*_a_ of 21.4 μM^−1^. To identify key residues that support a high-affinity IQ/ICDI interaction, we undertook systematic alanine substitution of the IQ domain and evaluated the effect on binding affinity in our FRET assay. Within IQ_1.3_, we substituted each residue with an alanine or, at loci where the wild-type channel featured an alanine, we replaced the residue with a threonine. For identification of each residue, the canonical isoleucine is assigned position 0. Application of our FRET assay to each mutated peptide identified three residues, Y[−5]A, F[−2]A, and F[+4]A, which severely perturbed the IQ/ICDI interaction (Fig. 1*E*, Fig. S1). Focusing on F[−2]A, FRET binding produced a shallower curve as compared with WT (Fig. 1*D*, *blue* *versus* *gray*), resulting in a *K*_a_ of 5.8 μM^−1^ (Fig. 1*E*, *blue*). Introducing this mutation into the chimeric channel resulted in a partial rescue of CDI (Fig. 1*F*), indicating that this interaction site is functionally relevant. However, the IQ domain substitutions Y[−5]A and F[+4]A, which also had a marked effect on *K*_a_, resulted in minimal CDI rescue (Fig. 1, *F* and *G*, Fig. S2). Importantly, these residues also serve as anchors for apoCaM binding to the Ca_V_1.3 IQ domain, resulting in weak baseline CDI even in the absence of the ICDI domain (Table S1) (13). Of note, this apoCaM effect may also underly the apparent increase in binding affinity demonstrated by several of the mutations, where a decrease in apoCaM binding would enhance the apparent binding of the IQ/ICDI regions due to altered competition with endogenous CaM.

In order to confirm the functional relevance of each binding loci identified, we turned to a previously described analysis known as individually transformed Langmuir (iTL) analysis (14). This approach allows us to rigorously correlate relative changes in binding with functional changes in CDI. As iTL was initially derived to evaluate the binding interfaces critical to CaM-mediated channel regulation (13, 14), we adjust the model to reflect the competitive binding scheme between CaM and ICDI (20). To do so, we first account for the ambiguity caused by binding sites, which are important for both apoCaM and ICDI binding. We therefore incorporate both the IQ domain’s intrinsic affinity for apoCaM (*K*_a-CaM_) and that for the ICDI segment (*K*_a-ICDI_), the competitive inhibitor, by adjusting our measured *K*_a-ICDI_ such that:

(1) $$K_{a-ICDI}^{corr}=\frac{K_{a-CaM}^{WT}}{K_{a-CaM}^{mut}}\cdot K_{a-ICDI}$$

Where $K_{a-CaM}^{WT}$ is the apoCaM binding affinity of WT PreIQ_3_-IQ-A_1.3_, and $K_{a-CaM}^{mut}$ represents the apoCaM binding affinity of each mutant peptide, values which were previously measured (13) and are listed in Table S1. This compensation remains valid provided that the local concentration of ICDI is much greater than *K*_d-ICDI_ ([ICDI]>> 1/*K*_a-ICDI_). With this adjustment made, CDI can be defined by a modified Langmuir function as follows:

(2) $$\frac{CDI}{CDI_{max}}\;=\;\frac{\left[apoCaM\right]\cdot K_{a-CaM}}{1\;+\;\left[apoCaM\right]\cdot K_{a-CaM}\;+\;\left[ICDI\right]\cdot K_{a-ICDI}^{corr}}$$

where *CDI* is the strength of CDI under endogenous levels of CaM; *CDI*_max_ is the CDI in saturating concentrations of CaM (Table S1); *K*_a-CaM_ is the association constant for apoCaM binding to PreIQ_3_-IQ-A_1.3_; [apoCaM] is the free apoCaM concentration in the cell; and [ICDI] is the effective local concentration of ICDI (see Supporting information for full derivation). Equation 2 predicts an inverse correlation between $K_{a-ICDI}^{corr}$ and relative *CDI*, which we can fit to our data (Fig. 1*H*). For channels containing WT IQ_1.3_ and ICDI_1.4_ domains, the strong binding between IQ and ICDI results in minimal CDI, as seen by the black data point on the plot. In contrast, Ca_V_1.3_Δ_ channels, which lack an ICDI domain such that $K_{a-ICDI}^{corr}=0$ by definition, display large CDI values (cyan). The F[−2]A mutation, which produced a partial restoration of CDI, resides in an intermediate position (blue). Overall, our data can be well fit by Equation 2 (Fig. 1*H*), confirming the functional relevance of the identified residues in a competitive model.

### Alanine scanning of the ICDI domain reveals complementary hotspots

**Figure 2:**
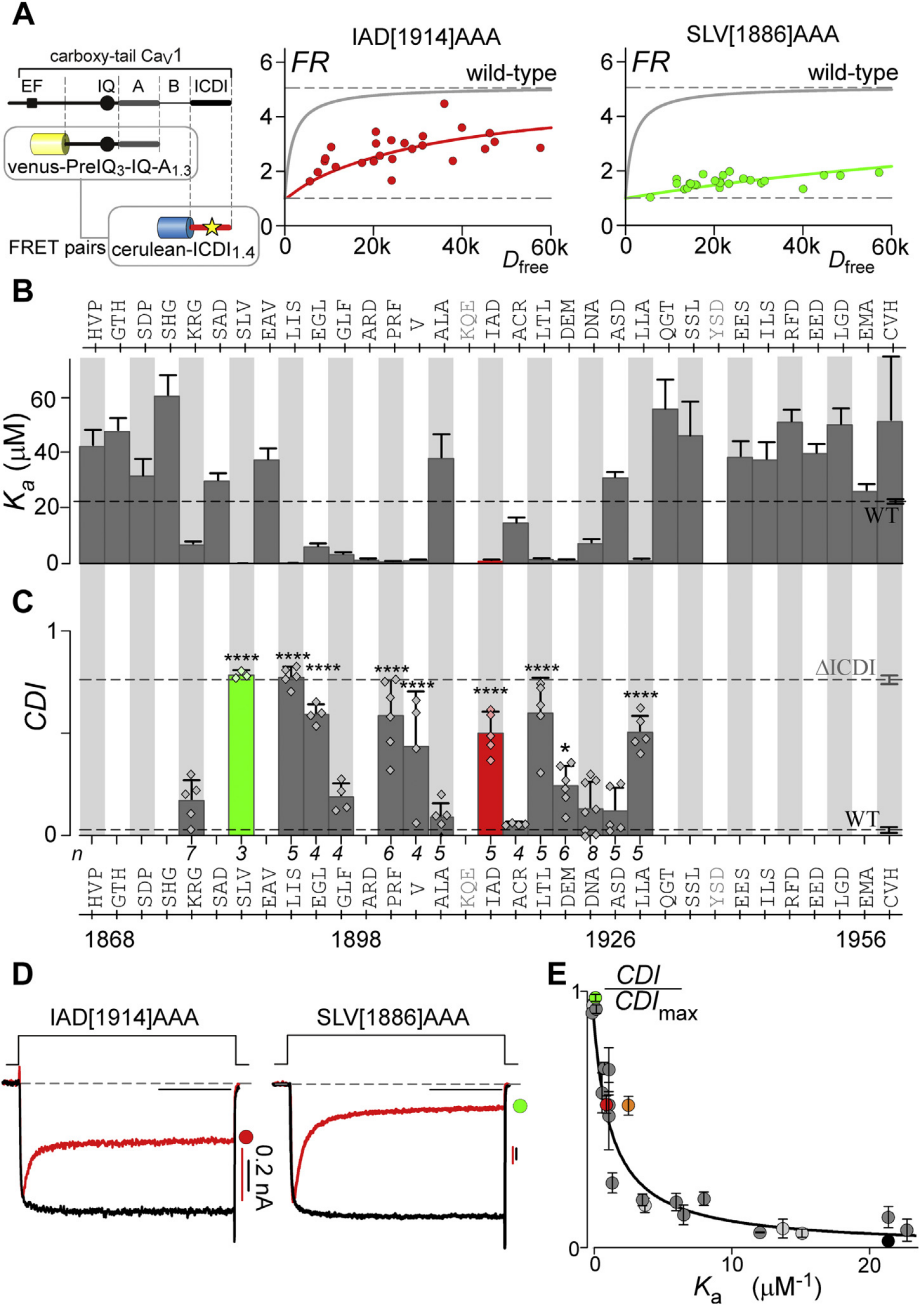
**Identifying the hotspots on ICDI**_**1.4**_**required for ICDI binding and function.***A*, FRET binding partners are displayed on the *left*, with the *star* indicating the locus of alanine mutations within ICDI_1.4_. Compared with WT (*gray line*), IAD[1914]AAA (*red*) and SLV[1886]AAA (*green*) significantly decreased binding affinity between the FRET pairs to differing degrees. *B*, summary of *K*_a_ values for Venus-PreIQ_3_-IQA_1.3_*versus* mutant Cerulean-ICDI_1.4_ peptides measured with FRET two-hybrid as in panel *A*. Alanine was systematically substituted three amino acids at a time into Cerulean-ICDI_1.4_, with the identity of the amino acids displayed on the *top of the bar graph*. Amino acids labeled in *gray* (KQE, YSD) were not evaluated due to poor expression. The *dashed line* indicates the WT value and the *colored bars* correspond to data in other panels of the figure here and throughout. Data are displayed ±SE. *C*, summary of CDI values measured for Ca_V_1.3_Δ/DCT1.4_ harboring mutations corresponding to mutations that produced large effects on binding affinity in *B*. *Gray dashed line* represents the robust CDI of the channel without ICDI_1.4_, while the *black dashed line* indicates the nominal CDI of WT Ca_V_1.3_Δ/DCT1.4_. Data are displayed ±SD (ANOVA summary analysis indicates a likelihood of significant difference at a level of *p* < 0.0001. Post-hoc Dunnett’s multiple comparisons *versus* WT indicated as ∗∗∗∗*p* < 0.0001; ∗*p* < 0.05). *D*, exemplar whole-cell recordings of Ca_V_1.3_Δ/DCT1.4_, demonstrating a significant restoration of CDI due to IAD[1914]AAA and near complete CDI due to SLV[1886]AAA. *E*, robust fit of CDI (*C*) and FRET binding data (*B*) to Equation 2, using identical parameters as in Figure 1*H*. *Light gray circles* indicate data from Figure 1, while *dark gray* and *colored circles* indicate mutations within ICDI corresponding to *B* and *C*, with *green* and *red circles* indicating exemplars shown in other panels for reference. The PKA phosphorylation site Ser1883 is plotted as the *orange circle*.

Having identified several critical residues within the IQ domain required for ICDI binding, we next probed the ICDI for critical determinates of binding to the IQ. In order to scan a more extensive segment of the channel, we made triple alanine substitutions for every three contiguous residues within the ICDI domain. The ICDI domain has previously been localized to amino acids 1868 to 1956 of the Ca_V_1.3 DCT (29, 32, 33). We therefore undertook our alanine scan on this segment of the channel. Importantly, this region includes the distal C-terminal regulatory domain (DCRD), which was previously identified as playing an important role in the ICDI-mediated inhibition of CDI (27, 31, 33, 47). Finally, we included a selective V[1907]A mutation, as this amino acid change has previously been shown to dramatically alter the function of ICDI (30). To evaluate the effect of these alanine substitutions on IQ/ICDI binding, we again utilized our FRET two-hybrid binding assay, pairing Venus-PreIQ_3_-IQ-A_1.3_ with Cerulean-ICDI_1.4_ (Fig. 2*A*, Fig. S3). Indeed, measured *K*_a_ values revealed multiple hotspots within the ICDI domain, with a wide range of binding affinities with the IQ containing peptide (Fig. 2*B*). Interestingly several mutations, largely toward either end of the peptide, appeared to modestly increase in *K*_a._ However, these changes may reflect a structural stabilization of the isolated ICDI peptide by promoting helicity of end regions and may not necessarily yield a corresponding change in intact channels. As such, we did not pursue further analysis of these mutants. The two mutation sites displayed in gray (KQE[1911]AAA and YSD[1941]AAA) were not evaluated as they failed to express. Notably, the effects of the hotspots on the ICDI domain were significantly larger than those observed within the IQ domain (Fig. 2*B* *versus* Fig. 1*E*).

In order to correlate loss of binding affinity with function, we measured the CDI of those mutations that exhibited a large change in binding affinity (Fig. 2*C*, Fig. S4). As predicted, mutations that resulted in a significant loss of IQ/ICDI binding also exhibited a corresponding restoration of CDI. Focusing on two examples, IAD[1914]AAA moderately reduced IQ/ICDI binding (Fig. 2, *A* and *B*, *red*), while introduction of the same mutations into our chimeric channel enabled a partial restoration of CDI (Fig. 2, *C* and *D*, *red*). On the other hand, SLV[1886]AAA displayed a drastic reduction in IQ/ICDI binding (Fig. 2, *A* and *B* *green*), and CDI was fully restored to the level seen in Ca_V_1.3_Δ_ (Fig. 2, *C* and *D* *green*). Importantly, all identified loci are well fit by our Langmuir function, such that the same set of Equation 2 parameters describes both the IQ region and ICDI (Fig. 2*E*). Of note, as mutations in ICDI do not affect the binding of apoCaM, the correction factor for *K*_a_ is no longer required, and $K_{a-ICDI}^{corr}=K_{a-ICDI}$. Having identified critical loci within ICDI, we note that one of these amino acids (S[1866]) has previously been identified as a phosphorylation site, which reduces the binding affinity of ICDI_1.4_ by about tenfold, while increasing CDI of Ca_V_1.3_Δ/DCT1.4_ (43). We therefore included the results of phosphorylation at this site in our analysis (Fig. 2*E*, *orange*). Indeed, phosphorylation of this amino acid results in a change in binding affinity, which correlates with CDI according to the same Langmuir function. Thus, we have identified numerous residues within ICDI_1.4_, which are critical determinants of a functional competition between ICDI and apoCaM for the IQ region of Ca_V_1.3.

### The role of the A region in the IQ/ICDI interaction

**Figure 3:**
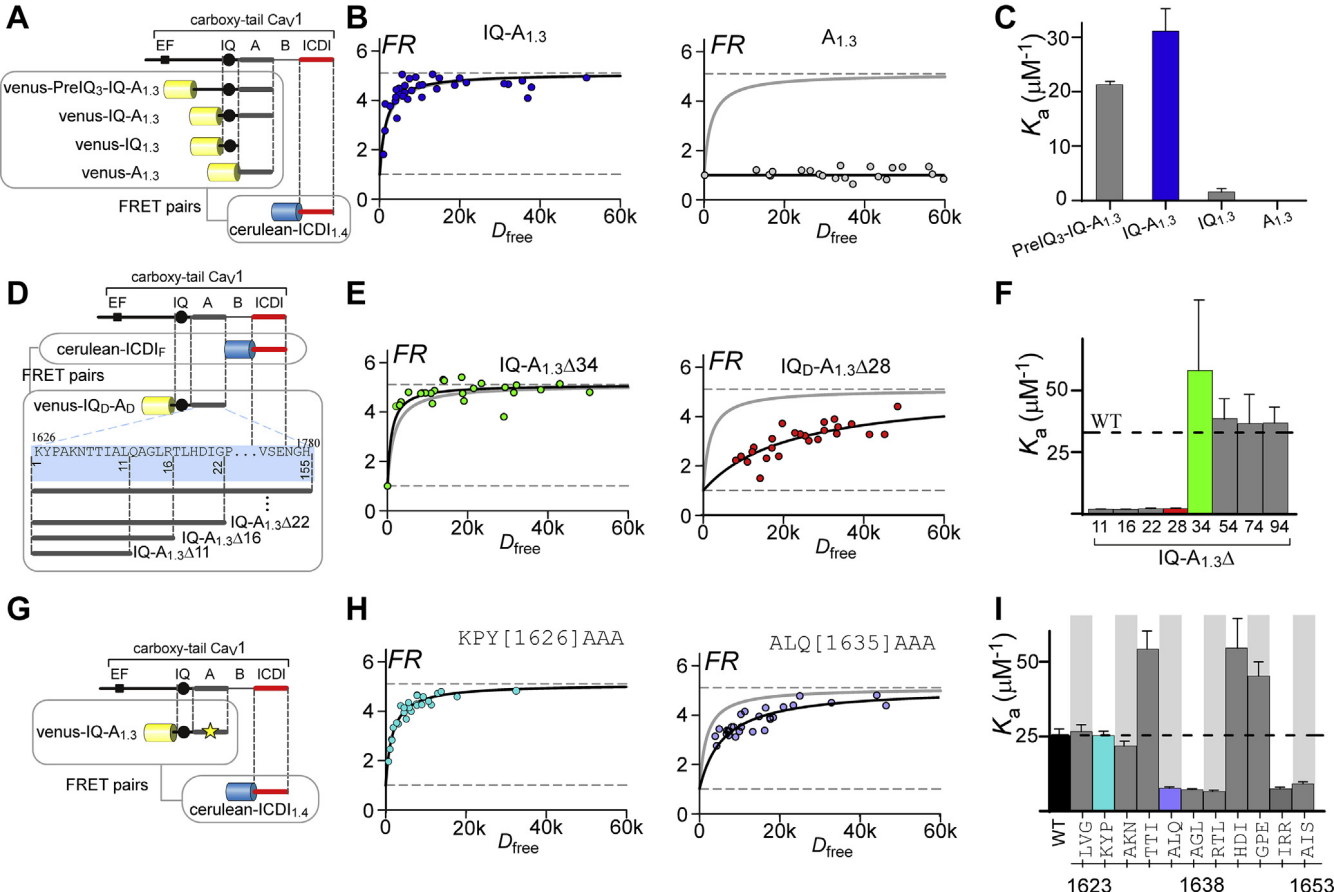
**Elucidating the role of the A region for ICDI interaction.***A*, cartoon depicting FRET interacting pairs for panels *B* and *C*. Portions of the PreIQ_3_-IQ-A_1.3_ were paired with ICDI_1.4_ to identify critical regions. *B*, IQ-A_1.3_ is sufficient to support robust FRET (*blue*), indicating that the pre-IQ_3_ region is not involved in the interaction. However, the A region alone is not sufficient to support binding (*open circles*). *C*, *K*_a_ values for each channel fragment indicate that neither IQ_1.3_ nor A_1.3_ is sufficient for robust binding with ICDI_1.4_; however, both regions are necessary as indicated by the robust binding of IQ-A_1.3_ with ICDI_1.4_. Data are displayed as mean ± SE here and throughout. *D*, cartoon depicting FRET interacting pairs for panels *E* and *F*. Cerulean-ICDI_1.4_ was paired with various truncations of Venus-PreIQ_3_-IQ-A_1.3_. The *blue box* shows the sequence of the A region and illustrates the truncation strategy. *E* and *F*, truncating up to 34 amino acids distal to the IQ region (Δ34) had nominal effects on binding (*green*); however, removal of six additional amino acids (Δ28) dramatically reduced binding affinity (*red*). *G*, cartoon depicting FRET interacting pairs for panels *H* and *I*. Alanine substitutions were made within the A region, as indicated by the *yellow star*. *H*, mutation of KPY[1626]AAA within the A region had no effect on binding (*cyan*), while ALQ[1635]AAA (*purple*) diminished the binding affinity between IQ-A_1.3_ and ICDI_1.4_. *I*, summary of *K*_a_ values for alanine mutations in the A region indicate multiple critical amino acids.

While our analysis identified several functionally relevant IQ domain loci, the impact of these mutations was far less than those identified within the ICDI (Fig. 1 *versus* Fig. 2). This suggests that additional regions outside the IQ domain may contribute to ICDI binding. In order to identify such regions, we generated truncated variations of our Venus-PreIQ_3_-IQ-A_1.3_ construct and paired them with Cerulean-ICDI_1.4_ in the FRET two-hybrid binding assays (Fig. 3*A*, Fig. S5). We began by removing the PreIQ_3,_ and found no change in FRET binding, indicating that all relevant interaction loci are contained within the IQ and A regions (Fig. 3*B*, *blue*). However, removal of the IQ domain, leaving only the A region intact, resulted in a complete loss of FRET binding (Fig. 3*B*, *open circles*). Likewise, the IQ region alone displayed no binding with ICDI (Fig. 3*C*), suggesting that both the IQ and the A region are necessary for interaction with ICDI. In order to further localize the critical interaction sites, we undertook successive truncation of the vernus-IQ-A_1.3_ peptide (Fig. 3*D*). FRET measurements demonstrated minimal effect of truncations up to 34 amino acids from the end of the A region, as demonstrated by the strong binding of Venus-IQ-A_1.3_Δ34 with ICDI_1.4_ (Fig. 3*E*, *green*). However, our next truncation, Venus-IQ-A_1.3_Δ28, exhibited a marked decrease in FRET binding (Fig. 3, *E* and *F*, *red*) Thus, the 34 residues immediately downstream of the IQ domain critically augment ICDI binding. Of note, this region includes the previously identified proximal C-terminal regulatory domain (PCRD), which is reported to play an important role in the ICDI interaction (27, 31, 33, 47). Having identified a subset of the A region, which is vital to ICDI binding, we again undertook systematic alanine substitutions, replacing each of the three contiguous residues with three alanine residues and undertook our FRET-2-hybrid binding assay (Fig. 3*G*). Disruptions in binding were identified as the result of a number of mutations, spanning both the previously identified PCRD region and a previously unidentified region upstream of this motif (Fig. 3, *H* and *I*, Fig. S6). Thus, both the IQ and the distal A region of the channel are required for high-affinity interaction with ICDI.

### The functional relevance of ICDI in Ca_V_1.2 channels

**Figure 4:**
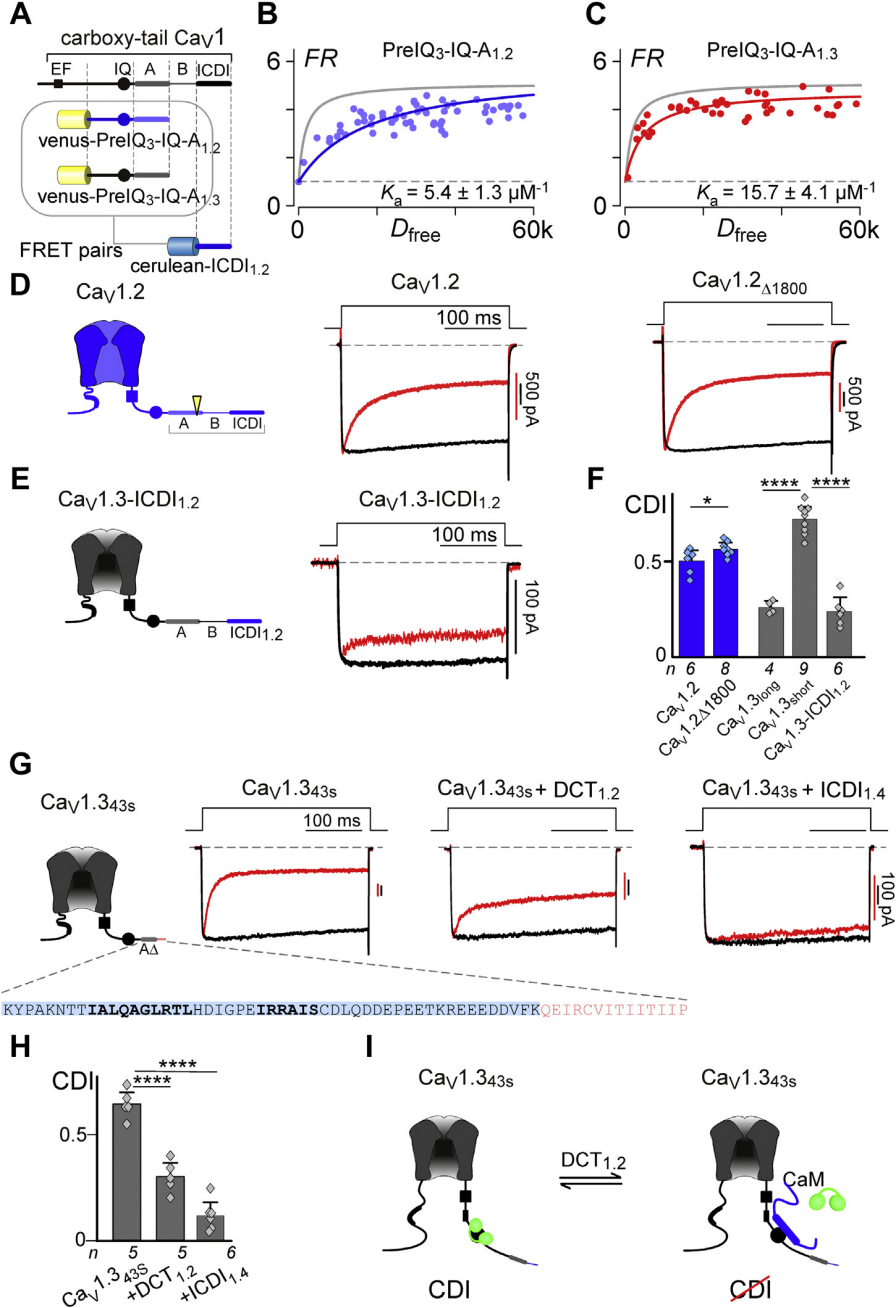
**Residual functionality of ICDI1.2 in modulating Ca**_**V**_**channels.***A*, cartoon depicting FRET interacting pairs for panels *B* and *C*. Venus-PreIQ_3_-IQ-A_1.2_ or Venus-PreIQ_3_-IQ-A_1.3_ was paired with Cerulean-ICDI_1.2_ in order to evaluate the role of the ICDI contained within Ca_V_1.2 channels. *B*, Venus-PreIQ_3_-IQ-A_1.2_ displays moderate binding with Cerulean-ICDI_1.2_ (*blue*) as compared with the robust binding between Venus-PreIQ_3_-IQ-A_1.3_ and Cerulean-ICDI_1.4_ which is reproduced as the *gray line* for comparison. *C*, Venus-PreIQ_3_-IQ-A_1.3_ is able to bind strongly with Cerulean-ICDI_1.2_. *D*, *Left*, channel cartoon with cleavage site indicated by the *yellow arrowhead*. *Right*, exemplar data demonstrating that truncation of Ca_V_1.2 at the cleavage site has a minor effect on CDI. *E*, *Left*, channel cartoon indicating introduction of ICDI_1.2_ into the Ca_V_1.3 channel backbone. *Right*, introduction of ICDI_1.2_ into Ca_V_1.3 channels causes a large decrease in CDI. *F*, average CDI data demonstrating a modest but statistically significant effect of ICDI_1.2_ on Ca_V_1.2 channels (*blue*), and a large effect on Ca_V_1.3, which is comparable to the native effect of ICDI_1.3_ contained within Ca_V_1.3_long_ (*gray*). Data are plotted as mean ± SD. (Ca_V_1.2: ∗*p* < 0.05 based on a Student’s *t*-test; Ca_V_1.3: ANOVA summary analysis indicates a likelihood of significant difference at a level of *p* < 0.0001. Post-hoc Dunnett’s multiple comparisons *versus* Ca_V_1.3_short_ indicated as ∗∗∗∗*p* < 0.0001.) *G*, *Left*, cartoon of human Ca_V_1.3_43S_, with the sequence of the end of the channel displayed below. This splice variant contains a portion of the A region (*blue highlight*), which contains the identified hotspots required for ICDI binding (*bold*), as well as a short sequence of unique amino acids prior to truncation of the channel (*pink*). *Right*, exemplar whole-cell patch clamp data demonstrates robust CDI in WT Ca_V_1.3_43S_, which is reduced when the predicted cleavage fragment of the human Ca_V_1.2 channel is coexpressed, or when ICDI_1.4_ is expressed as a peptide. *H*, average CDI effects for the channels described in panel *G*, DCT_1.2_ and ICDI_1.4_ both have significant effects on the CDI of Ca_V_1.3_43S_ (ANOVA summary analysis indicates a likelihood of significant difference at a level of *p* < 0.0001. Post-hoc Dunnett’s multiple comparisons *versus* Ca_V_1.3_43s_ indicated as ∗∗∗∗*p* < 0.0001; data are displayed ±SD). *I*, cartoon proposing a cross-channel effect of ICDI_1.2_, such that cleaved or independently transcribed DCT_1.2_ may modulate Ca_V_1.3_43S_ channels.

Similar to Ca_V_1.3 and Ca_V_1.4, Ca_V_1.2 channels also feature a highly homologous ICDI segment, argued to function as a channel inhibitor (33, 48) or as a transcriptional factor (41, 42). We therefore considered the impact of ICDI_1.2_ on both Ca_V_1.2 and Ca_V_1.3 channels. We interrogated the binding of Cerulean-ICDI_1.2_ with Venus-PreIQ_3_-IQ-A_1.2_ *via* FRET two-hybrid (Fig. 4, *A* and *B*) and found that the interaction is significantly weaker than the prototypic Venus-PreIQ_3_-IQ-A_1.2_ and Cerulean-ICDI_1.4_ interaction (Fig. 4*B* *versus* Fig. 1*D*). However, when paired with Venus-PreIQ_3_-IQ-A_1.3_, binding with ICDI_1.2_ is significantly larger, and only about half that of the strong binding of ICDI_1.4_ (Fig. 4*C*). Thus, it appears that ICDI_1.2_ is poised to have a larger effect in the context of Ca_V_1.3 channels as compared with its native channel backbone. Nonetheless, the limited binding observed between Cerulean-ICDI_1.2_ and Venus-PreIQ_3_-IQ-A_1.2_ prompted us to evaluate the possibility of a functional role for ICDI_1.2_ within Ca_V_1.2 channels. Interestingly, truncation of Ca_V_1.2 at the known carboxy-tail cleavage site (49) for this channel (Ca_V_1.2_Δ1800_) resulted in a minimal, yet statistically significant (*p* ≤ 0.05), increase in CDI (Fig. 4, *D* and *F*, Fig. S7). Next, to test the effect of ICDI_1.2_ on Ca_V_1.3 channels, we replaced the native ICDI_1.3_ of Ca_V_1.3_long_ channels with ICDI_1.2_. Indeed, the loss of CDI surpassed that of Ca_V_1.2 channels (Fig. 4*E*), as predicted based on the stronger PreIQ_3_-IQ-A_1.3_/ICDI_1.2_ interaction (Fig. 4*C*). In fact, the CDI exhibited by Ca_V_1.3-ICDI_1.2_ channels was not statistically different than the CDI measured in the native Ca_V_1.3_long_ splice variant (Fig. 4*F*).

Multiple studies have shown that the DCT of Ca_V_1.2, containing ICDI_1.2_, exists as a peptide within neurons and cardiomyocytes, either due to proteolysis (33, 49) or as a result of alternative transcriptional initiation sites (41). Moreover, it has previously been demonstrated that ICDI domains can exert their effects on L-type channels when expressed as separate peptides (27). We therefore sought to recreate the potential interaction of select Ca_V_1.3 channel variants with the DCT of Ca_V_1.2. To begin, we choose the human Ca_V_1.3_43S_ splice variant of Ca_V_1.3, as these channels terminate just past the A region and thus lack an inherent ICDI module (27). In addition, the inclusion of the A region within Ca_V_1.3_43S_ has been demonstrated to be important for ICDI binding, both in functional experiments done by others (27) and in our alanine scan of the A region (Fig. 3). We therefore generated the proteolytic product of human Ca_V_1.2 channels (DCT_1.2_) and evaluated the effect of this peptide on the CDI of Ca_V_1.3_43S_. Indeed, consistent with previous studies (27), coexpression of DCT_1.2_ significantly reduced the CDI of Ca_V_1.3_43S_ (Fig. 4*G*, Fig. S7). For comparison, we also coexpressed these channels with ICDI_1.4_ expressed as a peptide, which we have shown has a *K*_a_ about double that of ICDI_1.2_ (Fig. 4*C* *versus* Fig. 1*D*). Indeed, ICDI_1.4_ results in an even larger CDI deficit when expressed with Ca_V_1.3_43S_ (Fig. 4*, G* and *H*). Thus, the proteolytically cleaved DCT_1.2_ is well poised to exert a significant modulation of select Ca_V_1.3 channel variants, such that the ambient concentrations of CaM and DCT_1.2_ are able to tune the CDI of Ca_V_1.3_43S_ channels in a competitive manner (Fig. 4*I*).

## Discussion

CaM regulation of Ca_V_ channels is vital to normal physiology and thus has been the subject of intense study (8, 10, 11, 17, 50, 51, 52). The competitive mechanism of ICDI within select Ca_V_1 channels forms a basis with which CaM regulation can be tuned (12, 20, 32). Numerous processes designed to modulate this regulation include splice variation, RNA editing, variations in ambient CaM concentration, and phosphorylation (12, 13, 18, 36, 43). Identification of critical loci involved in this regulation is therefore key to understanding how CaM regulation may vary in different physiological and pathological states. As such, in-depth residue-level analysis not only reveals interfaces utilized by cells to tune channel regulation, but may offer targets in the search for novel regulators of the channel, which may have therapeutic benefit. In particular, the dramatically different efficacy of ICDI across channel subtypes may offer the possibility of subtype selective drug targeting, which remains challenging for Ca_V_1 channels.

Given the importance of understanding these interactions within the carboxy-tail of Ca_V_1 channels, we quantified the structure–function relationship of these interactions using a variant of previously described iTL analysis (14). This provided a major advantage in that the quantitative agreement of our results with Equation 2 demonstrates that each identified locus is functionally relevant. This overcomes a common limitation of binding assays between channel fragments, which may identify sites that are inaccessible or inconsequential in the context of the holochannel. Moreover, by fitting to a specific Langmuir curve, we can distinguish mutations that may alter channel function through ancillary mechanisms such as transduction or altered folding of the channel. Thus, in addition to identifying critical loci, our results confirm the competitive mechanism described for ICDI modulation of CDI.

A number of previous studies have identified regions within the carboxy tail of Ca_V_1 channels, which are critical to the competitive mechanism of ICDI inhibition. Among these are the PCRD and DCRD regions, which were identified within Ca_V_1.2 channels as potential interaction sites such that the DCRD region of the proteolytically cleaved Ca_V_1.2 DCT may interact with the PCRD on the channel *via* electrostatic interaction with the negatively charged amino acids (33). Homologous regions within Ca_V_1.3 and Ca_V_1.4 were later shown to be important for ICDI regulation and for the interaction with modular peptides (27, 30, 31, 33, 47). In this study, the PCRD resides within the A region and overlaps with the identified locus of critical amino acids required for high-affinity binding between the IQ-A and ICDI. Interestingly, these critical amino acids were identified both within the PCRD and upstream of the motif, arguing for a larger interacting region, which is highly conserved across Ca_V_ channels (Fig. S8). However, our binding assay also demonstrated that the A region, in itself, is insufficient for high-affinity binding, but also requires the upstream IQ region (Fig. 3*A*). This fits with previous findings in which neutralization of the PCRD arginines was not sufficient to prevent the ICDI inhibition of CDI (31), pointing to the existence of additional interaction loci. In a similar manner, our scan of the ICDI region validated the importance of the DCRD, while also identifying numerous critical interacting loci upstream of the motif (Fig. S8). Thus, this study has expanded our knowledge of the important interactions required for ICDI inhibition, providing a comprehensive map of the critical loci within the carboxy tail.

The impact of ICDI in Ca_V_1.3 and Ca_V_1.4 channels has been well recognized; however, its role in Ca_V_1.2 has been uncertain. It has been demonstrated that the truncation of Ca_V_1.2 results in increased current density, altered voltage dependence of channel activation, and disrupted targeting of the channel to the membrane (33, 53, 54); however, no impact on CDI has been reported. Here, we find that the impact of ICDI_1.2_ within Ca_V_1.2 channels is minimal, allowing cleavage of this DCT region without significant disruption of CDI. Yet ICDI_1.2_ is capable of causing significant disruption of CDI in the context of Ca_V_1.3 (Fig. 4, *E*–*I*) (27). This selective effect of ICDI_1.2_ on Ca_V_1.3 channels is intriguing as it represents a difficult to achieve discrimination between Ca_V_1.2 and Ca_V_1.3 (55, 56). The strong homology between these two channels has resulted in challenges to dissecting the contribution of each channel to the function of cells, which express both channel subtypes, and hinders therapeutic options for neuropsychiatric disorders, which may benefit from blockade of Ca_V_1.3 (55). As such, the interface defined in this study between the IQ-A region and ICDI may represent a promising interface with which to selectively target Ca_V_1.3.

The DCT of Ca_V_1.2 is routinely cleaved in neurons and myocytes, with the cleavage product able to either remain associated with the channel (33) or translocate to the nucleus (41). Our FRET binding data would argue that this association between Ca_V_1.2 and the DCT may be relatively weak, leaving DCT_1.2_ available to other binding partners. Moreover, it has been shown that an alternate start site exists within the carboxy tail of Ca_V_1.2, such that alternative transcription of Ca_V_1.2 will produce a DCT peptide containing a calcium channel-associated transcription regulator (CCAT) (41). Importantly, ICDI would be intact within this peptide, providing an additional source of DCT_1.2_ within cells. In addition, CCAT has been shown to localize to the cytosol in a Ca^2+^-dependent manner, providing a source of ICDI in proximity to the membrane, which can be tuned by activity (41). Our FRET binding analysis (Fig. 4*A*) suggests that the Ca_V_1.2 DCT is capable of binding upstream calmodulatory elements in Ca_V_1.2, albeit weakly. Functional analysis, however, suggests only minimal effects of this segment on Ca_V_1.2 CDI. By comparison, the Ca_V_1.3_43S_ channel variant contains all the elements required for high-affinity binding with DCT_1.2_ and exhibits functional inhibition of CDI (Fig. 4*G*), consistent with previous studies (27). Moreover, Ca_V_1.3_43S_ is widely expressed in the brain, accounting for up to 39% of transcripts, and is often expressed within the same neuron type as Ca_V_1.2 (27). Thus, it seems likely that DCT_1.2_ may interact with this channel, altering the normally robust CDI. It is interesting to note, however, that while DCT_1.2_ is poised to modulate some Ca_V_1.3 channels, the same cannot be said of DCT_1.3_. Not only is the IQ-A region of Ca_V_1.2 suboptimal for binding to ICDI, but there is little evidence that Ca_V_1.3 is cleaved in neurons (31). Thus, this cross-channel modulation may be unidirectional. Finally, since Ca_V_1.2 and Ca_V_1.3 often exist within the same neuron, this mode of cross-channel modulation may represent an important method for tuning CDI in different regions of the brain.

## Experimental procedures

### Molecular biology

The rat brain Ca_V_1.3 α1 subunit (in pcDNA6) corresponds to AF370009.1 (57) and was incorporated to the mammalian expression plasmid pCDNA6 (Invitrogen) (20). This plasmid features a unique *Bgl*II restriction site at a locus corresponding to ∼450 amino acids upstream of the IQ domain and a unique *Xba*I site after the stop codon, which were used for generation of mutant and chimeric plasmids as descried below. The Ca_V_1.2 α1 subunit (in pGW) is identical to rabbit NM001136522 (58), and the Ca_V_1.4 channel (in pcDNA3) is the human clone corresponding to NP005174.2. Ca_V_1.3_Δ/DCT1.4_ was made by fusing with the DCT of the Ca_V_1.4 α1 subunit to the Ca_V_1.3 α1 subunit (truncated after the IQ domain), as previously described (20). Ca_V_1.3_43S_ was made by PCR amplification of the channel segment between the *Bgl*II site and IQ domain with the appendage of amino acids unique to this splice variant (27). The PCR product was then inserted into the channel *via* the *Bgl*II/*Xba*I sites.

FRET constructs were fluorescent-tagged (either Venus or Cerulean) using similar strategies as previously described (45). Briefly, Venus and Cerulean fluorophores (a kind gift from Dr Steven Vogel, NIH) were subcloned into the pcDNA3 vector *via* unique *Kpn*I and *Not*I sites. The PCR-amplified channel peptides, as described in Liu *et al.*, (20) were then cloned *via* unique *Not*I and *Xba*I sites. Mutations were introduced into the channel or FRET construct *via* PCR amplification or overlap extension PCR.

### Transfection of HEK293 cells

For electrophysiology experiments, HEK293 cells were cultured on 10-cm plates, and channels were transiently transfected by a calcium phosphate protocol (10). We applied 8 μg of plasmid DNA encoding the desired pore forming α1 subunit, as well as 8 μg of β_2A_ (M80545) and 8 μg of rat α_2δ_ (NM012919.2) subunits along with 3 μg of SV40 T antigen. For microscope-based FRET assays, HEK293 cells cultured on 3.5-cm culture dishes with integral No. 0 glass coverslip bottoms (*In Vitro* Scientific) were transiently transfected using polyethylenimine (PEI) reagent (Polysciences).

### Whole-cell patch clamp recordings

Whole-cell recordings were obtained using an Axopatch 200A amplifier (Axon Instruments). Electrodes were pulled from borosilicate glass capillaries (World Precision Instruments), with 1 to 3 MΩ resistances, which were in turn compensated for series resistance by >60%. Currents were low-pass filtered at 2 kHz before digital acquisition at five times the frequency. A P/8 leak subtraction protocol was used. The internal solution contained (in mM): CsMeSO3, 114; CsCl, 5; MgATP, 4; HEPES (pH 7.4), 10; and BAPTA (1,2-bis(*o*-aminophenoxy)ethane- *N*,*N*,*N’*,*N’*-tetraacetic acid), 10; at 295 mOsm adjusted with CsMeSO3. The bath solution contained (in mM): TEA-MeSO3, 102; HEPES (pH 7.4), 10; CaCl2 or BaCl2, 40; at 305 mOsm adjusted with TEA-MeSO3. Data was analyzed using custom Matlab scripts. Inactivation was quantified as the ratio of current remaining after 300 ms (current amplitude measured at 300 ms divided by peak current amplitude) in either Ca^2+^ or Ba^2+^ (*r*_300_). CDI was then quantified as the *r*_300_ in Ca^2+^ subtracted from the *r*_300_ in Ba^2+^, measured at 10 mV for Ca_V_1.3 channels, and 30 mV for Ca_V_1.2.

### FRET optical imaging

FRET two-hybrid experiments were performed on an inverted microscope as described (45, 46). The bath solution was a Tyrode’s solution composed of (in mM): NaCl, 138; KCl, 4; MgCl2, 1; HEPES (pH 7.4), 10; CaCl2, 2; at 305 mOsm adjusted with glucose. Background fluorescent signals were measured from cells without expression of the fluorophores and subtracted from cells expressing the fluorophores. Concentration-dependent spurious FRET was subtracted from the raw data prior to binding-curve analysis (45, 46). Cerulean (59) and Venus (60) were used as the donor and acceptor fluorescent proteins instead of eCFP and eYFP, as their optical properties provided more robust and stable FRET signals. Acceptor-centric measurements of FRET were obtained with the 3^3^-FRET algorithm (45, 46), in which the effective FRET efficiency (*E*_EFF_) and FRET ratio (*FR*) are defined as:

(3) $$E_{\text{Eff}}=E\times A_{\text{b}}=\left(FR-1\right)\left[\varepsilon_{\text{Ven}\left(440\;\text{nm}\right)}/\varepsilon_{\text{Cer}\left(440\;\text{nm}\right)}\right]$$

where *E* is the FRET efficiency of a donor–acceptor pair, *A*_*b*_ is the fraction of acceptor molecules bound by a donor, and ${\text{ε}}_{\text{Ven}\left(440\;\text{nm}\right)}/{\text{ε}}_{\text{Cer}\left(440\;\text{nm}\right)}$ is the approximate molar extinction coefficients of Cerulean and Venus, which was measured as 0.08 on our setup. Intensity measurements at each wavelength were taken from individual cells such that variable expression across the cells enabled population of a binding curve. Binding curves were analyzed using GraphPad software (Prism), providing relative *K*_d_ values and standard error based on an unbiased fit to the data. These relative K_d_ values were then calibrated according to a previously determined calibration factor (13, 20) and converted to *K*_a_ = 1/*K*_d_. Importantly, the previous calibration factor determined for our setup utilized CFP/YFP FRET pairs. In order to account for the difference in FR using Cerulean and Venus, we determined the relative *K*_d_ for multiple peptides using both the fluorophore pairs and found that the two data sets differed by a factor of 1.8, which we incorporated into the calibration factor.

## Data availability

All data is contained within the article.

## Supporting information

This article contains supporting information (13, 14, 20, 33, 43, 49).

## Conflict of interest

The authors declare that they have no conflicts of interest with the contents of this article.

## References

[1] Harvey R.D., Hell J.W. (2013). CaV1.2 signaling complexes in the heart. J. Mol. Cell. Cardiol..

[2] Gutierrez L.M., Brawley R.M., Hosey M.M. (1991). Dihydropyridine-sensitive calcium channels from skeletal muscle. I. Roles of subunits in channel activity. J. Biol. Chem..

[3] Striessnig J., Koschak A., Sinnegger-Brauns M.J., Hetzenauer A., Nguyen N.K., Busquet P., Pelster G., Singewald N. (2006). Role of voltage-gated L-type Ca2+ channel isoforms for brain function. Biochem. Soc. Trans..

[4] Lipscombe D., Helton T.D., Xu W. (2004). L-type calcium channels: The low down. J. Neurophysiol..

[5] McDonald T.F., Pelzer S., Trautwein W., Pelzer D.J. (1994). Regulation and modulation of calcium channels in cardiac, skeletal, and smooth muscle cells. Physiol. Rev..

[6] Ghosh D., Syed A.U., Prada M.P., Nystoriak M.A., Santana L.F., Nieves-Cintron M., Navedo M.F. (2017). Calcium channels in vascular smooth muscle. Adv. Pharmacol..

[7] Liang H., DeMaria C.D., Erickson M.G., Mori M.X., Alseikhan B.A., Yue D.T. (2003). Unified mechanisms of Ca^2+^ regulation across the Ca^2+^ channel family. Neuron.

[8] Pitt G.S., Zuhlke R.D., Hudmon A., Schulman H., Reuter H., Tsien R.W. (2001). Molecular basis of calmodulin tethering and Ca2+-dependent inactivation of L-type Ca2+ channels. J. Biol. Chem..

[9] Alseikhan B.A., DeMaria C.D., Colecraft H.M., Yue D.T. (2002). Engineered calmodulins reveal the unexpected eminence of Ca2+ channel inactivation in controlling heart excitation. Proc. Natl. Acad. Sci. U. S. A..

[10] Peterson B.Z., DeMaria C.D., Adelman J.P., Yue D.T. (1999). Calmodulin is the Ca^2+^ sensor for Ca2+ -dependent inactivation of L- type calcium channels. Neuron.

[11] Zuhlke R.D., Pitt G.S., Deisseroth K., Tsien R.W., Reuter H. (1999). Calmodulin supports both inactivation and facilitation of L-type calcium channels. Nature.

[12] Adams P.J., Ben-Johny M., Dick I.E., Inoue T., Yue D.T. (2014). Apocalmodulin itself promotes ion channel opening and Ca(2+) regulation. Cell.

[13] Bazzazi H., Ben Johny M., Adams P.J., Soong T.W., Yue D.T. (2013). Continuously tunable Ca(2+) regulation of RNA-edited CaV1.3 channels. Cell Rep..

[14] Ben Johny M., Yang P.S., Bazzazi H., Yue D.T. (2013). Dynamic switching of calmodulin interactions underlies Ca2+ regulation of CaV1.3 channels. Nat. Commun..

[15] Splawski I., Timothy K.W., Decher N., Kumar P., Sachse F.B., Beggs A.H., Sanguinetti M.C., Keating M.T. (2005). Severe arrhythmia disorder caused by cardiac L-type calcium channel mutations. Proc. Natl. Acad. Sci. U. S. A..

[16] Splawski I., Timothy K.W., Sharpe L.M., Decher N., Kumar P., Bloise R., Napolitano C., Schwartz P.J., Joseph R.M., Condouris K., Tager-Flusberg H., Priori S.G., Sanguinetti M.C., Keating M.T. (2004). Ca(V)1.2 calcium channel dysfunction causes a multisystem disorder including arrhythmia and autism. Cell.

[17] Griessmeier K., Cuny H., Roetzer K., Griesbeck O., Harz H., Biel M., Wahl-Schott C. (2009). Calmodulin is a functional regulator of Ca_V_1.4 l-type Ca^2+^ channels. J. Biol. Chem..

[18] Haeseleer F., Williams B., Lee A. (2016). Characterization of C-terminal splice variants of Cav1.4 Ca2+ channels in human retina. J. Biol. Chem..

[19] Yang P.S., Alseikhan B.A., Hiel H., Grant L., Mori M.X., Yang W., Fuchs P.A., Yue D.T. (2006). Switching of Ca^2+^-dependent inactivation of Ca_V_1.3 channels by calcium binding proteins of auditory hair cells. J. Neurosci..

[20] Liu X., Yang P.S., Yang W., Yue D.T. (2010). Enzyme-inhibitor-like tuning of Ca(2+) channel connectivity with calmodulin. Nature.

[21] Yang P.S., Johny M.B., Yue D.T. (2014). Allostery in Ca(2)(+) channel modulation by calcium-binding proteins. Nat. Chem. Biol..

[22] Shaltiel L., Paparizos C., Fenske S., Hassan S., Gruner C., Rotzer K., Biel M., Wahl-Schott C.A. (2012). Complex regulation of voltage-dependent activation and inactivation properties of retinal voltage-gated Cav1.4 L-type Ca2+ channels by Ca2+-binding protein 4 (CaBP4). J. Biol. Chem..

[23] Campiglio M., Coste de Bagneaux P., Ortner N.J., Tuluc P., Van Petegem F., Flucher B.E. (2018). STAC proteins associate to the IQ domain of CaV1.2 and inhibit calcium-dependent inactivation. Proc. Natl. Acad. Sci. U. S. A..

[24] Niu J., Dick I.E., Yang W., Bamgboye M.A., Yue D.T., Tomaselli G., Inoue T., Ben-Johny M. (2018). Allosteric regulators selectively prevent Ca(2+)-feedback of CaV and NaV channels. Elife.

[25] Polster A., Dittmer P.J., Perni S., Bichraoui H., Sather W.A., Beam K.G. (2018). Stac proteins suppress Ca(2+)-dependent inactivation of neuronal l-type Ca(2+) channels. J. Neurosci..

[26] Wong King Yuen S.M., Campiglio M., Tung C.C., Flucher B.E., Van Petegem F. (2017). Structural insights into binding of STAC proteins to voltage-gated calcium channels. Proc. Natl. Acad. Sci. U. S. A..

[27] Bock G., Gebhart M., Scharinger A., Jangsangthong W., Busquet P., Poggiani C., Sartori S., Mangoni M.E., Sinnegger-Brauns M.J., Herzig S., Striessnig J., Koschak A. (2011). Functional properties of a newly identified C-terminal splice variant of Cav1.3 L-type Ca2+ channels. J. Biol. Chem..

[28] Tan G.M., Yu D., Wang J., Soong T.W. (2012). Alternative splicing at C-terminus of CaV1.4 calcium channel modulates calcium-dependent inactivation, activation potential and current density. J. Biol. Chem..

[29] Wahl-Schott C., Baumann L., Cuny H., Eckert C., Griessmeier K., Biel M. (2006). Switching off calcium-dependent inactivation in L-type calcium channels by an autoinhibitory domain. Proc. Natl. Acad. Sci. U. S. A..

[30] Lieb A., Scharinger A., Sartori S., Sinnegger-Brauns M.J., Striessnig J. (2012). Structural determinants of CaV1.3 L-type calcium channel gating. Channels (Austin).

[31] Singh A., Gebhart M., Fritsch R., Sinnegger-Brauns M.J., Poggiani C., Hoda J.C., Engel J., Romanin C., Striessnig J., Koschak A. (2008). Modulation of voltage- and Ca^2+^-dependent gating of Ca_V_1.3 L-type calcium channels by alternative splicing of a C-terminal regulatory domain. J. Biol. Chem..

[32] Singh A., Hamedinger D., Hoda J.C., Gebhart M., Koschak A., Romanin C., Striessnig J. (2006). C-terminal modulator controls Ca^2+^-dependent gating of Ca_V_1.4 L-type Ca^2+^ channels. Nat. Neurosci..

[33] Hulme J.T., Yarov-Yarovoy V., Lin T.W., Scheuer T., Catterall W.A. (2006). Autoinhibitory control of the CaV1.2 channel by its proteolytically processed distal C-terminal domain. J. Physiol..

[34] Christel C.J., Cardona N., Mesirca P., Herrmann S., Hofmann F., Striessnig J., Ludwig A., Mangoni M.E., Lee A. (2012). Distinct localization and modulation of Cav1.2 and Cav1.3 L-type Ca2+ channels in mouse sinoatrial node. J. Physiol..

[35] Mangoni M.E., Couette B., Bourinet E., Platzer J., Reimer D., Striessnig J., Nargeot J. (2003). Functional role of L-type Cav1.3 Ca2+ channels in cardiac pacemaker activity. Proc. Natl. Acad. Sci. U. S. A..

[36] Huang H., Tan B.Z., Shen Y., Tao J., Jiang F., Sung Y.Y., Ng C.K., Raida M., Kohr G., Higuchi M., Fatemi-Shariatpanahi H., Harden B., Yue D.T., Soong T.W. (2012). RNA editing of the IQ domain in Ca(v)1.3 channels modulates their Ca(2)(+)-dependent inactivation. Neuron.

[37] Bezprozvanny I. (2009). Calcium signaling and neurodegenerative diseases. Trends Mol. Med..

[38] Hurley M.J., Brandon B., Gentleman S.M., Dexter D.T. (2013). Parkinson's disease is associated with altered expression of CaV1 channels and calcium-binding proteins. Brain.

[39] Hoda J.C., Zaghetto F., Koschak A., Striessnig J. (2005). Congenital stationary night blindness type 2 mutations S229P, G369D, L1068P, and W1440X alter channel gating or functional expression of Ca(v)1.4 L-type Ca2+ channels. J. Neurosci..

[40] Strom T.M., Nyakatura G., Apfelstedt-Sylla E., Hellebrand H., Lorenz B., Weber B.H., Wutz K., Gutwillinger N., Ruther K., Drescher B., Sauer C., Zrenner E., Meitinger T., Rosenthal A., Meindl A. (1998). An L-type calcium-channel gene mutated in incomplete X-linked congenital stationary night blindness. Nat. Genet..

[41] Gomez-Ospina N., Tsuruta F., Barreto-Chang O., Hu L., Dolmetsch R. (2006). The C terminus of the L-type voltage-gated calcium channel Ca(V)1.2 encodes a transcription factor. Cell.

[42] Schroder E., Byse M., Satin J. (2009). L-type calcium channel C terminus autoregulates transcription. Circ. Res..

[43] Sang L., Dick I.E., Yue D.T. (2016). Protein kinase A modulation of CaV1.4 calcium channels. Nat. Commun..

[44] Doering C.J., Hamid J., Simms B., McRory J.E., Zamponi G.W. (2005). Cav1.4 encodes a calcium channel with low open probability and unitary conductance. Biophys. J..

[45] Erickson M.G., Alseikhan B.A., Peterson B.Z., Yue D.T. (2001). Preassociation of calmodulin with voltage-gated Ca(2+) channels revealed by FRET in single living cells. Neuron.

[46] Erickson M.G., Liang H., Mori M.X., Yue D.T. (2003). FRET two-hybrid mapping reveals function and location of L-type Ca2+ channel CaM preassociation. Neuron.

[47] Liu N., Yang Y., Ge L., Liu M., Colecraft H.M., Liu X. (2017). Cooperative and acute inhibition by multiple C-terminal motifs of L-type Ca(2+) channels. Elife.

[48] Fuller M.D., Emrick M.A., Sadilek M., Scheuer T., Catterall W.A. (2010). Molecular mechanism of calcium channel regulation in the fight-or-flight response. Sci. Signal..

[49] Hulme J.T., Konoki K., Lin T.W., Gritsenko M.A., Camp D.G., Bigelow D.J., Catterall W.A. (2005). Sites of proteolytic processing and noncovalent association of the distal C-terminal domain of CaV1.1 channels in skeletal muscle. Proc. Natl. Acad. Sci. U. S. A..

[50] Chaudhuri D., Issa J.B., Yue D.T. (2007). Elementary mechanisms producing facilitation of Cav2.1 (P/Q-type) channels. J. Gen. Physiol..

[51] Kim J., Ghosh S., Nunziato D.A., Pitt G.S. (2004). Identification of the components controlling inactivation of voltage-gated Ca^2+^ channels. Neuron.

[52] Lee A., Zhou H., Scheuer T., Catterall W.A. (2003). Molecular determinants of Ca(2+)/calmodulin-dependent regulation of Ca(v)2.1 channels. Proc. Natl. Acad. Sci. U. S. A..

[53] Gerhardstein B.L., Gao T., Bunemann M., Puri T.S., Adair A., Ma H., Hosey M.M. (2000). Proteolytic processing of the C terminus of the alpha(1C) subunit of L-type calcium channels and the role of a proline-rich domain in membrane tethering of proteolytic fragments. J. Biol. Chem..

[54] Gao T., Bunemann M., Gerhardstein B.L., Ma H., Hosey M.M. (2000). Role of the C terminus of the alpha 1C (CaV1.2) subunit in membrane targeting of cardiac L-type calcium channels. J. Biol. Chem..

[55] Striessnig J., Ortner N.J., Pinggera A. (2015). Pharmacology of L-type calcium channels: Novel drugs for old targets?. Curr. Mol. Pharmacol..

[56] Huang H., Ng C.Y., Yu D., Zhai J., Lam Y., Soong T.W. (2014). Modest CaV1.342-selective inhibition by compound 8 is beta-subunit dependent. Nat. Commun..

[57] Xu W., Lipscombe D. (2001). Neuronal Ca(V)1.3alpha(1) L-type channels activate at relatively hyperpolarized membrane potentials and are incompletely inhibited by dihydropyridines. J. Neurosci..

[58] Wei X.Y., Perez-Reyes E., Lacerda A.E., Schuster G., Brown A.M., Birnbaumer L. (1991). Heterologous regulation of the cardiac Ca2+ channel alpha 1 subunit by skeletal muscle beta and gamma subunits. Implications for the structure of cardiac L-type Ca2+ channels. J. Biol. Chem..

[59] Rizzo M.A., Springer G.H., Granada B., Piston D.W. (2004). An improved cyan fluorescent protein variant useful for FRET. Nat. Biotechnol..

[60] Nagai T., Ibata K., Park E.S., Kubota M., Mikoshiba K., Miyawaki A. (2002). A variant of yellow fluorescent protein with fast and efficient maturation for cell-biological applications. Nat. Biotechnol..

