# Supplementary material for: The molecular basis of the inhibition of CaV1 calcium-dependent inactivation by the distal carboxy tail
Source: J Biol Chem. 2021 Mar 2;296:100502. doi: 10.1016/j.jbc.2021.100502 (PMC8054141; doi:10.1016/j.jbc.2021.100502)
Supplement: Supplemental Figures S1–S8 and Table S1 [file mmc1.pdf]

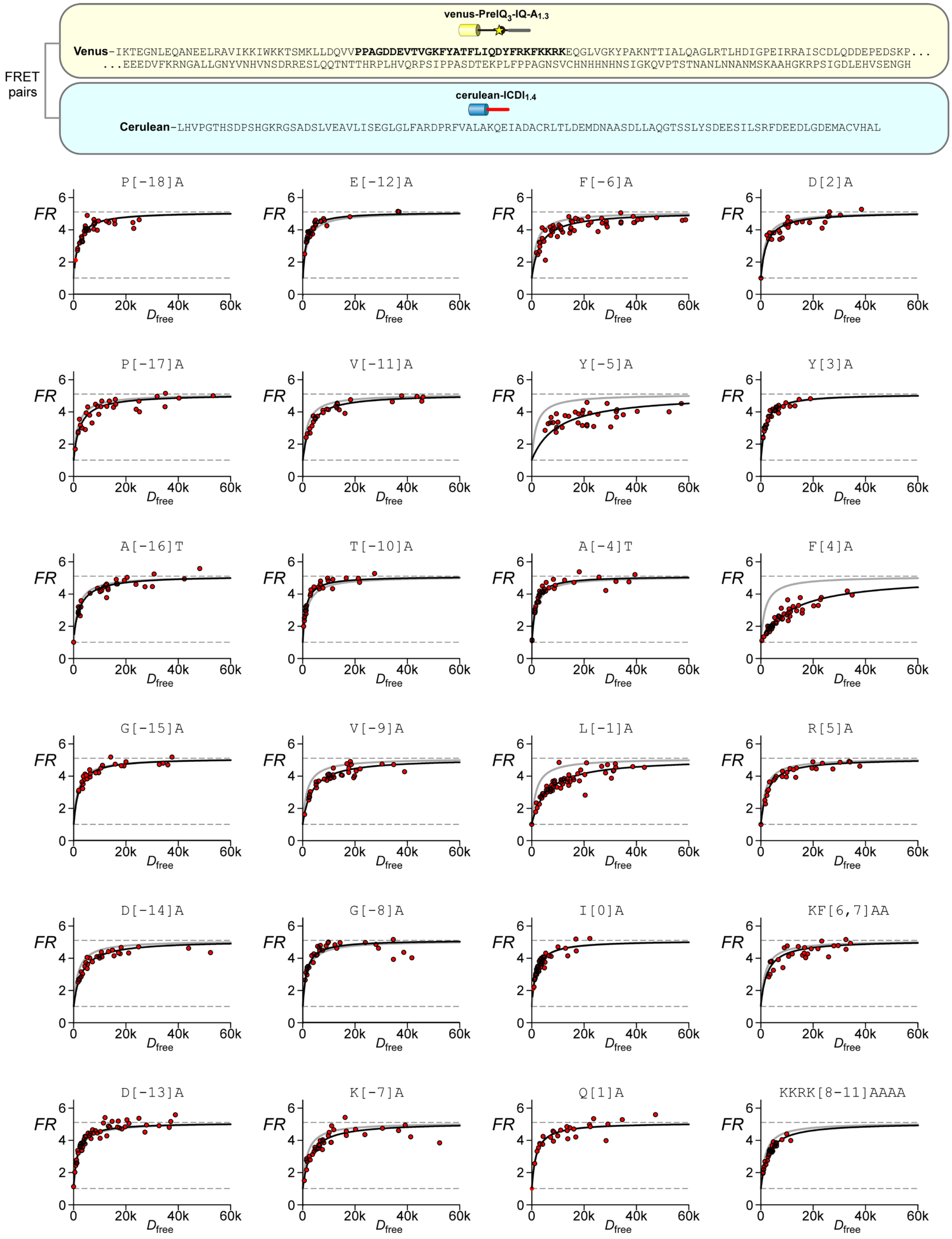

Figure S1: Top**:** Full sequence for the FRET 2-hybrid peptides used in main text Figure 1, with IQ region in bold. Below: FRET 2-hybrid binding curves for each mutation summarized in main text Figure 1E.

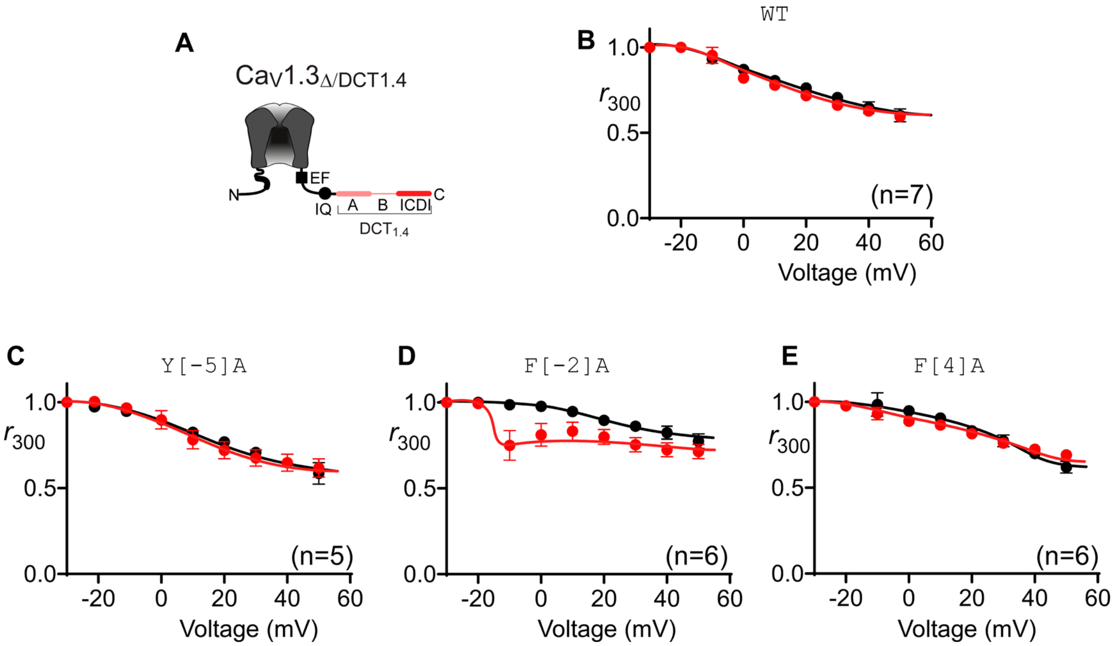

Figure S2: Full CDI dataset showing inactivation in Ca^{2+} (red) versus Ba^{2+} (black) as a function of voltage for WT and mutant $Ca_V1.3_{\Delta/DCT1.4}$, corresponding to data displayed in Figure 1G of the main text. Data is displayed ± SEM.

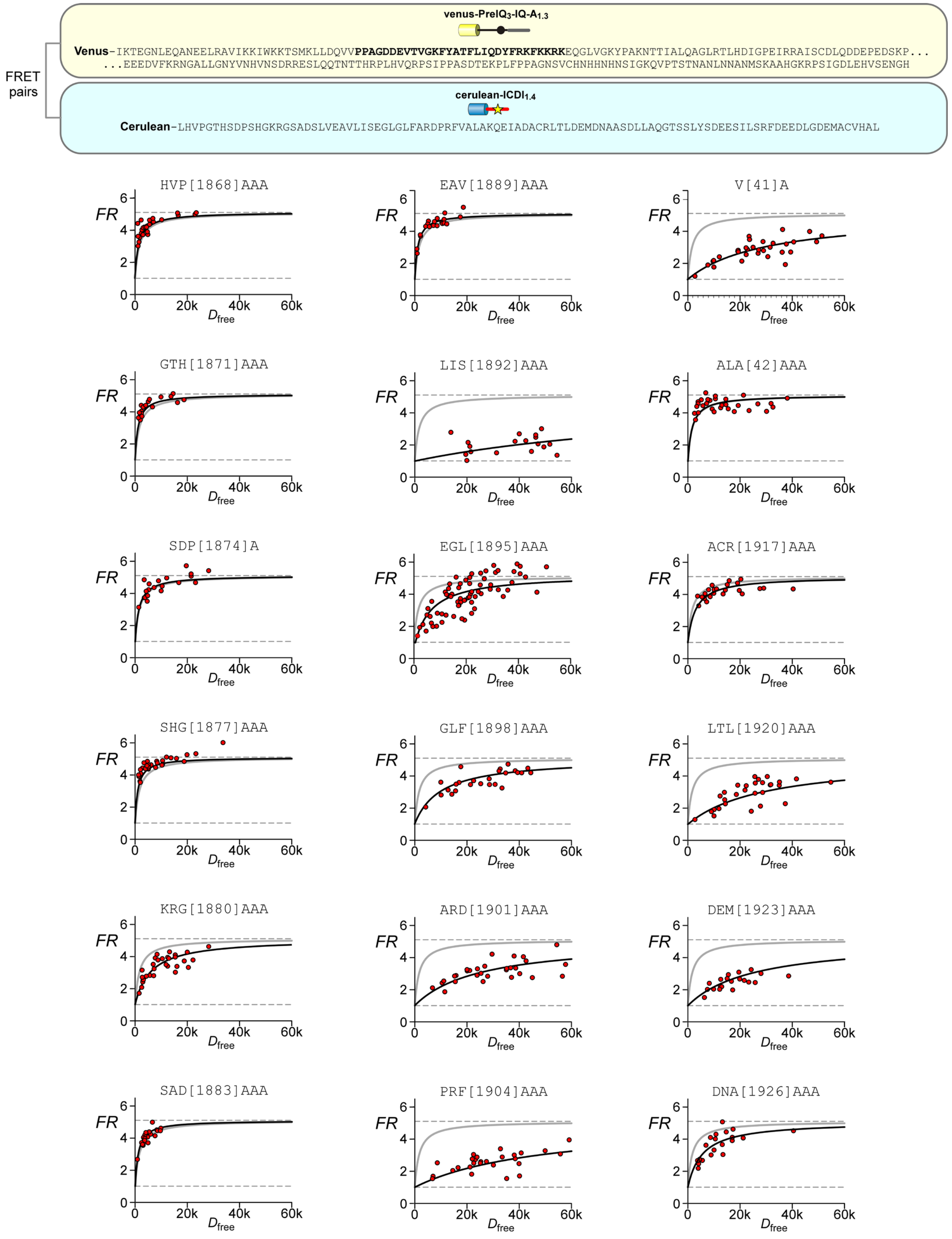

Figure S3: Top: Full sequence for the FRET 2-hybrid peptides used in main text Figure 2, with IQ region in bold. Below: FRET 2-hybrid binding curves for each mutation summarized in main text Figure 2B.

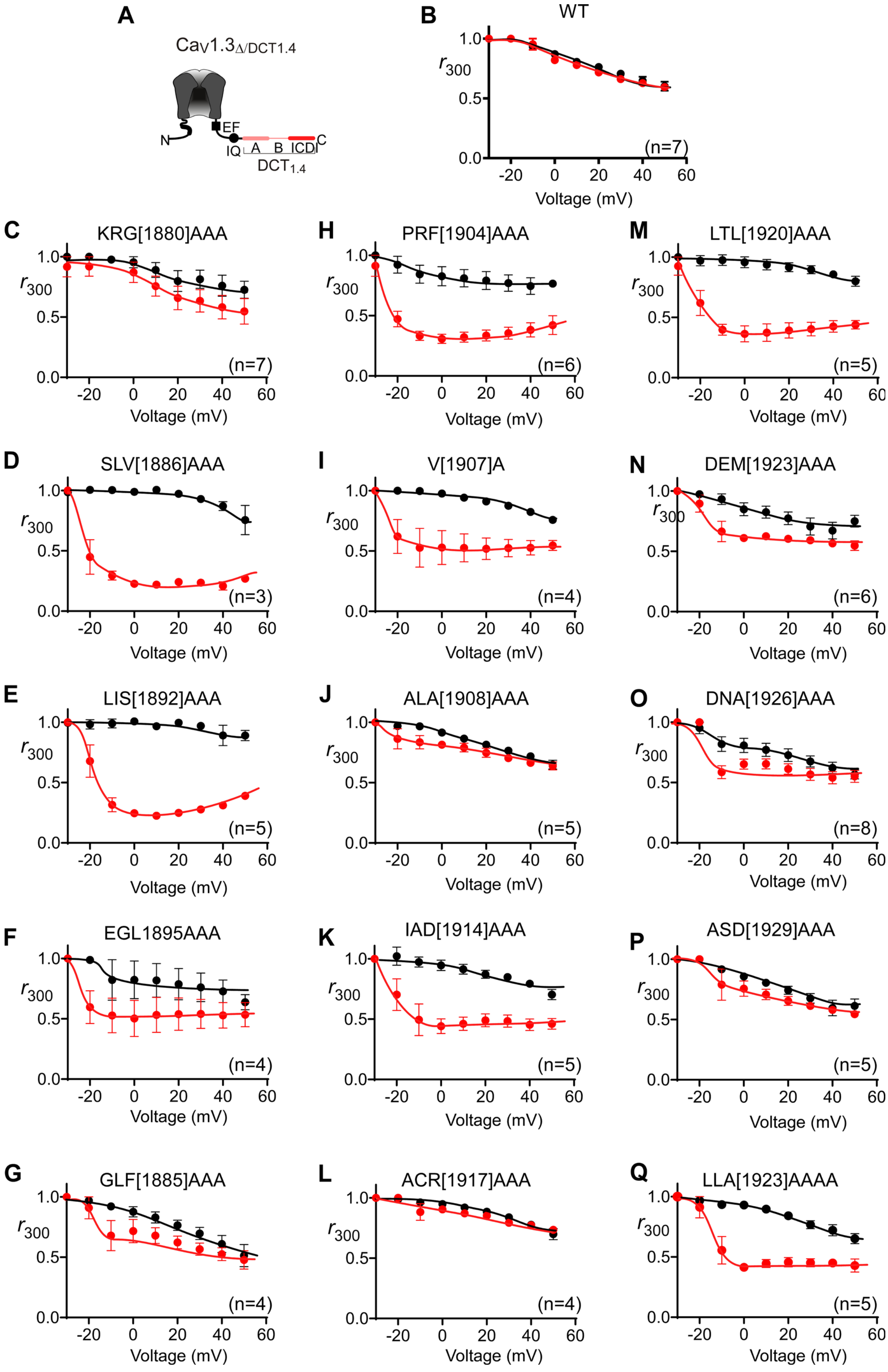

Figure S4: Full CDI dataset showing inactivation in Ca^{2+} (red) versus Ba^{2+} (black) as a function of voltage for WT and mutant $Ca_V1.3_{\Delta/DCT1.4}$, corresponding to data displayed in Figure 2C of the main text. Data is displayed ± SEM.

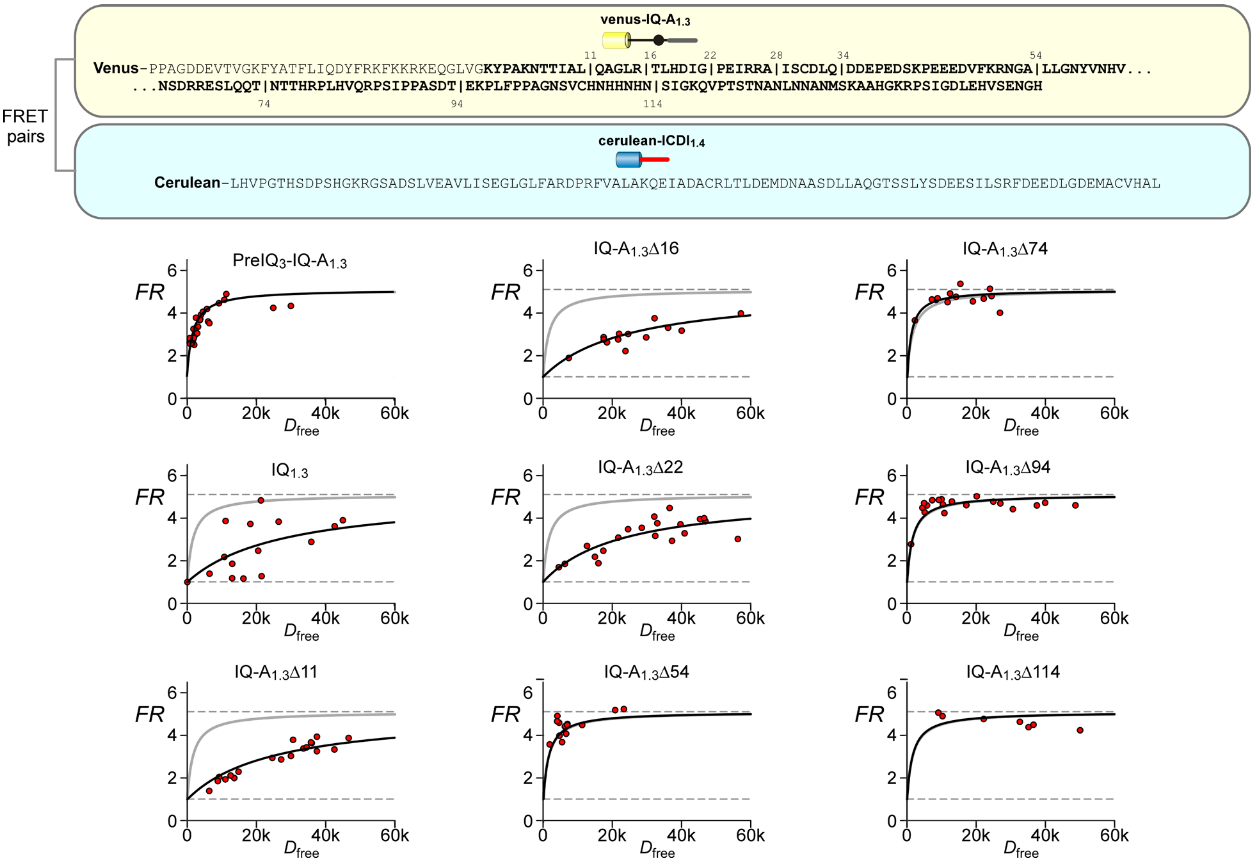

Figure S5: Top**:** Full sequence for the FRET 2-hybrid peptides used in main text Figure 3A-F, with A region in bold, and deletion sites marked by '|'. Below: FRET 2-hybrid binding curves for each deletion summarized in main text Figure 2C, F.

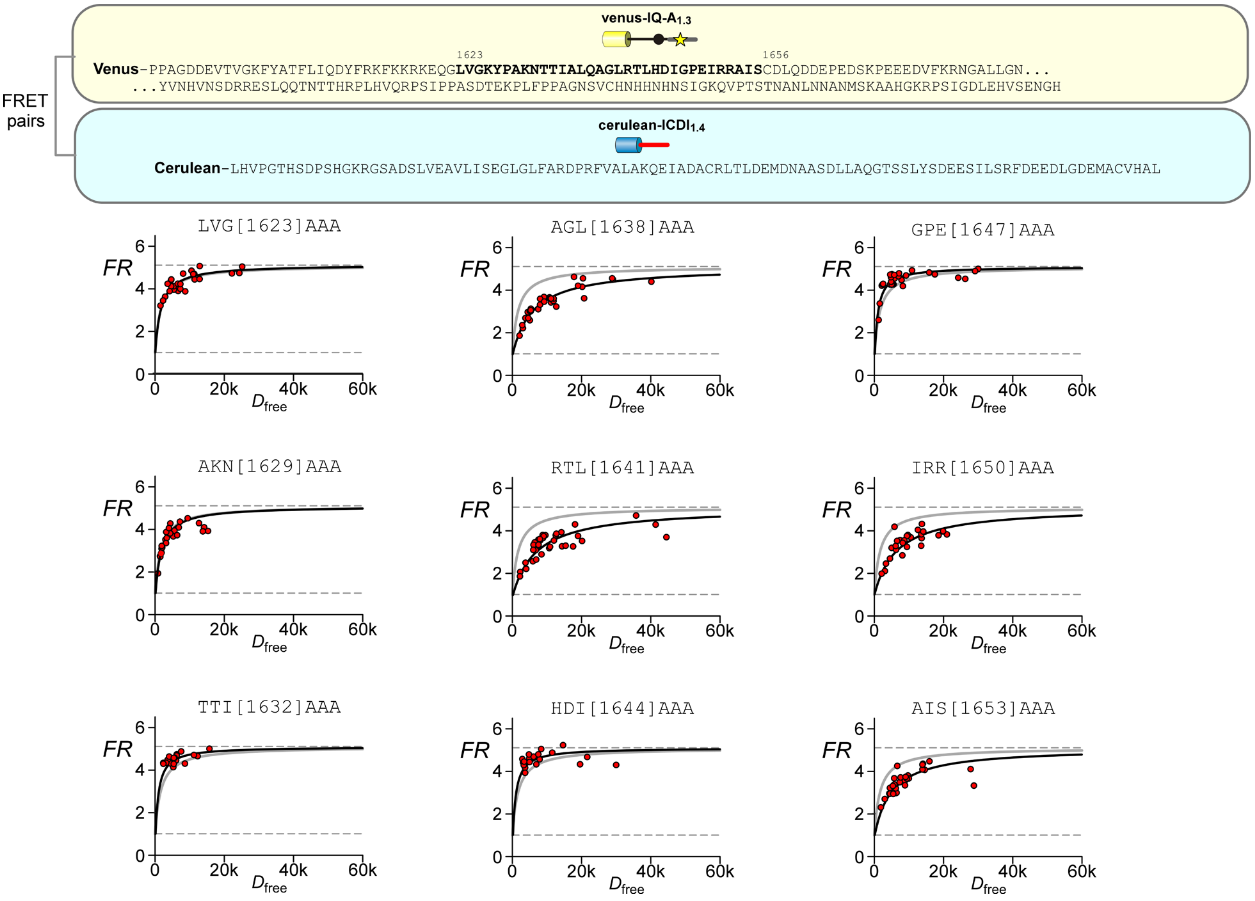

Figure S6: Top**:** Full sequence for the FRET 2-hybrid peptides used in main text Figure 3G-I, with the identified critical portion of the A region in bold. Below: FRET 2-hybrid binding curves for each mutation summarized in main text Figure 2I.

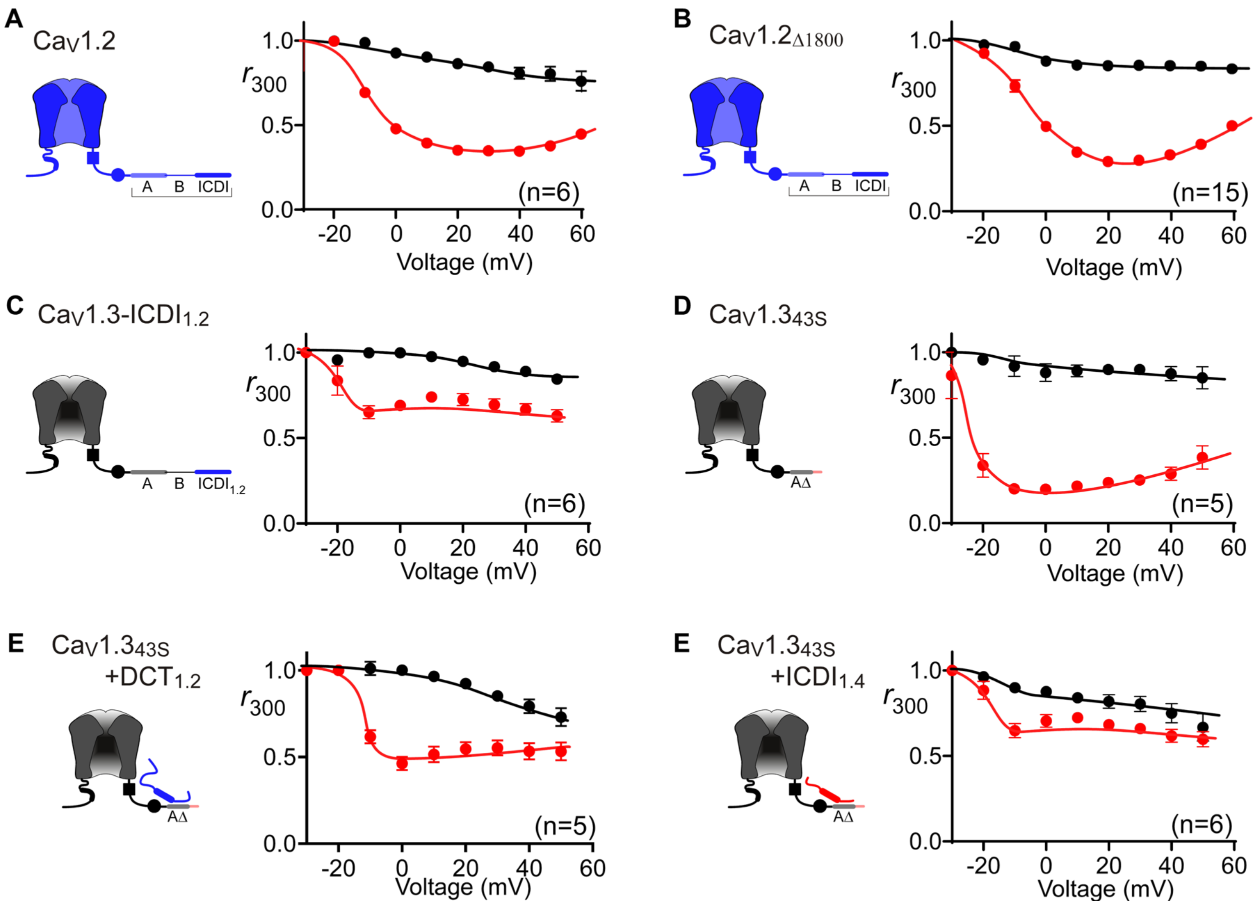

Figure S7: Full CDI dataset showing inactivation in Ca^{2+} (red) versus Ba^{2+} (black) as a function of voltage, corresponding to data displayed in Figure 4D-G of the main text. Data is displayed ± SEM.

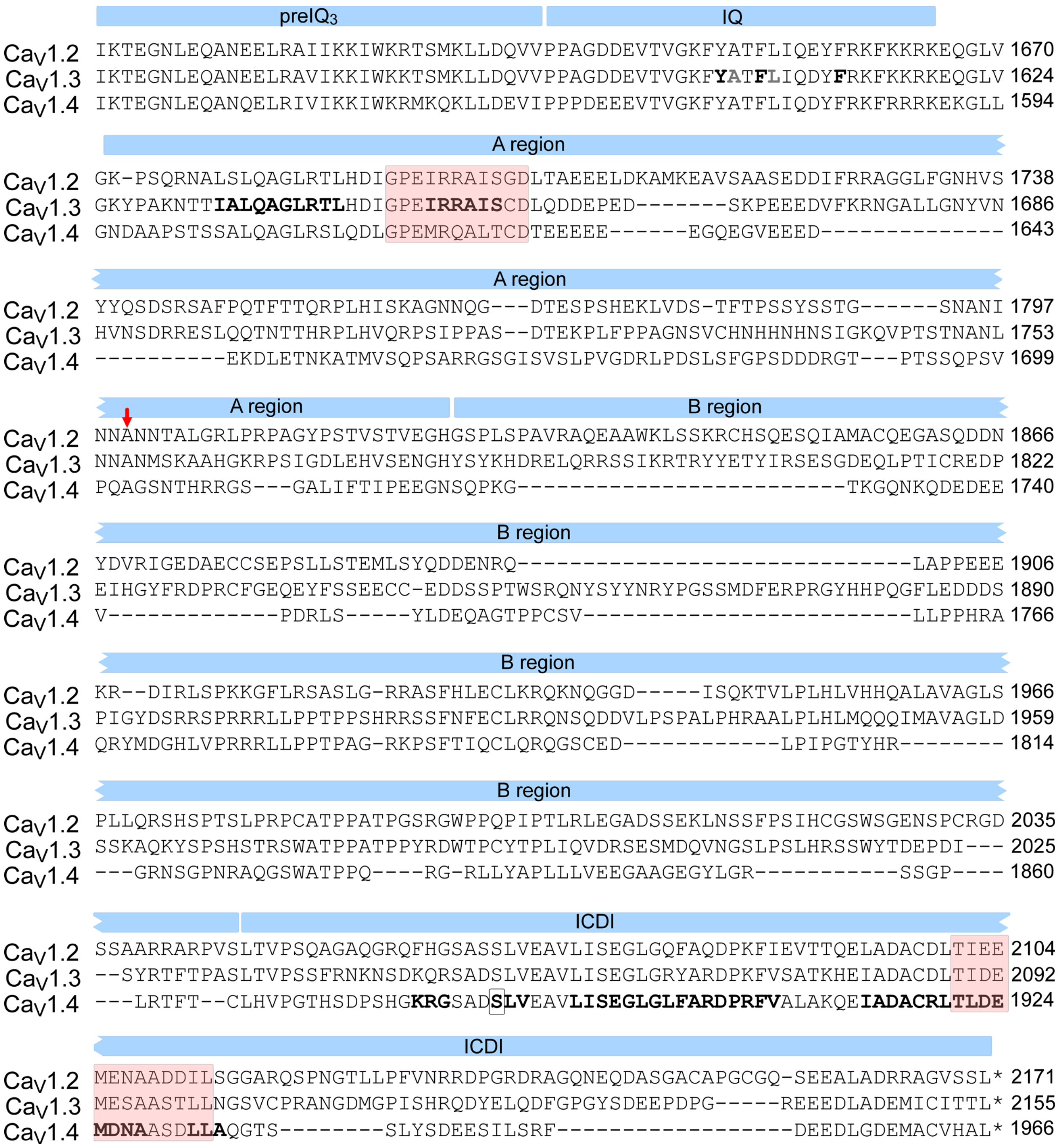

Figure S8: Alignment of the DCT of rabbit $Ca_V1.2$, rat $Ca_V1.3$ and human $Ca_V1.4$ corresponding to the channels used in main text figures 1-3. Relevant regions as defined in the text are marked in blue. Previously reported PCRD and DCRD regions (1) are highlighted in pink; the known cleavage site for the DCT is indicated by the red arrow (1,2); previously identified phosphorylation site is outlined by a black box (3). Critical residues identified in this study are in bold.

$Ca_V1.3$ IQ mutation	$K_{a, Ch}$	CDI_{max}
WT	16.346	0.808
F[4]A	3.269	0.717
F[-2]A	22.231	0.747
Y[-5]A	7.192	0.802

Table S1: Parameters used to fit equation 1 to data in Fig. 1H. Values were originally measured in (4).

Derivation of equations 1 and 2

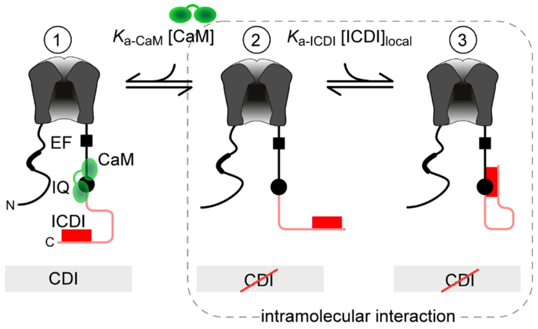

Figure S9: State transition diagram depicting competitive binding of CaM versus ICDI to the channel proximal carboxy-terminus. Only CaM bound conformation undergoes CDI.

A general framework for iTL analysis has been previously established for calmodulatory systems of any arbitrary architecture(4-6). Accordingly, perturbations of the equilibrium constants for a single transition within a general state-transition diagram follows a Langmuir relation with the equilibrium probability of the CDI state that is observed experimentally. We here develop a specific formulation that incorporates the competitive nature of ICDI and CaM interactions with the proximal carboxy-terminus. In the simplest case, consider a three-state model as shown in Figure S9. State 1 corresponds to a configuration where apoCaM is bound to the proximal carboxy-terminus, thus allowing the channel to undergo maximal CDI given by CDI_{max}. State 2 corresponds to the channel configuration where neither CaM nor ICDI occupies the proximal carboxy-terminus. Lastly, state 3 depicts a configuration where the ICDI domain occupies the proximal carboxy-tail, thus preventing CaM interaction. As both state 2 and 3 lack apoCaM, channels in these configurations are incapable of undergoing CDI. Given this framework, the steady-state probability of occupying the configurations 1 and 3 are given by:

$$P_1 = K_{a-CaM} \cdot [\mathrm{CaM}]_{\mathrm{free}} \cdot P_2 \quad \text{and} \quad P_3 = K_{a-ICDI} \cdot [\mathrm{ICDI}]_{\mathrm{local}} \cdot P_2 \qquad \text{-- (1)}$$

Here, [CaM] refers to the free ambient concentration of apoCaM, while $[\mathrm{ICDI}]_{local}$ denotes the effective local concentration of ICDI, as this domain is covalently attached to channel with a long linker encoded by the B-region. As $P_1 + P_2 + P_3 = 1$, the probability of occupying state 1 can be simplified as:

$$P_1 = \frac{K_{a-CaM} \cdot [\mathrm{CaM}]_{\mathrm{free}}}{K_{a-ICDI} \cdot [\mathrm{ICDI}]_{\mathrm{local}} + 1 + K_{a-CaM} \cdot [\mathrm{CaM}]_{\mathrm{free}}} \qquad \text{-- (2)}$$

As only state 1 is capable of eliciting CDI (yielding CDI_{max}), the effective CDI incorporating competition between ICDI and CaM is given by,

$$CDI = CDI_{\max} \cdot P_1 + 0 \cdot P_2 + 0 \cdot P_3 = CDI_{\max} \cdot P_1 \qquad \text{-- (3)}$$

Combining equations 2 and 3 yield:

$$\frac{CDI}{CDI_{\max}} = \frac{K_{a-CaM} \cdot [\mathrm{CaM}]_{\mathrm{free}}}{K_{a-ICDI} \cdot [\mathrm{ICDI}]_{\mathrm{local}} + 1 + K_{a-CaM} \cdot [\mathrm{CaM}]_{\mathrm{free}}} \qquad \text{-- (4)}$$

Importantly, the effective local concentration of the ICDI domain (i.e. $[\mathrm{ICDI}]_{\mathrm{local}}$) is a constant and incorporates entropic factors resulting from the spatial arrangement of ICDI domain within the channel complex. The free concentration of apoCaM may also be assumed to be relatively constant within HEK cells, as these cells contain an abundance of endogenous apoCaM binding targets that effectively serve to buffer for cytosolic free CaM.

Given this framework, consider the effect of mutations within the ICDI domain. As the apoCaM binding interface of $\mathrm{Ca_V1}$ channels is localized within the IQ, and upstream segments of the proximal carboxy-tail, mutations in the ICDI domain are unlikely to directly perturb CaM

association. Thus, ICDI mutations would largely affect only the equilibrium constant for the transition between state 2 and state 3 ($K_{a\text{-}ICDI}$).

Thus, by grouping all the constant terms, Eq 4 may be rewritten as,

$$\frac{CDI}{CDI_{\max}} = \frac{K_{a-CaM} \cdot [\mathrm{CaM}]_{\mathrm{free}} / [\mathrm{ICDI}]_{\mathrm{local}}}{K_{a-ICDI} + (1 + K_{a-CaM} \cdot [\mathrm{CaM}]_{\mathrm{free}}) / [\mathrm{ICDI}]_{\mathrm{local}}} \quad \text{-- (5)}$$

Importantly, as the affinity of apoCaM preassociation is quite strong (<100 nM) and as the local concentration of ICDI domain is high given its spatial localization within the channel complex, $(1 + K_{a-CaM} \cdot [\mathrm{CaM}]_{\mathrm{free}})/[\mathrm{ICDI}]_{\mathrm{local}} \sim K_{a-CaM} \cdot [\mathrm{CaM}]_{\mathrm{free}}/[\mathrm{ICDI}]_{\mathrm{local}} = \Gamma$ Thus, Eq. 5 reduces to

$$\frac{CDI}{CDI_{\max}} = \frac{\Gamma}{K_{a-ICDI} + \Gamma} \quad \text{-- (6)}$$

Experimentally, $K_{a\text{-}ICDI}$ is proportional to the relative binding affinities for each mutant obtained from FRET experiments. Thus, CDI/CDI_{max} follows a hyperbolic relationship with the relative strength of ICDI interaction with the IQ-A domains.

Similarly, mutations in the IQ domain may disrupt proximal carboxy-tail interaction with either ICDI or with CaM. Thus, both Γ and $K_{a\text{-}ICDI}$ in Eq. 6 may vary depending on the precise mutation. To compare effects of IQ mutations and ICDI mutations on a single curve, one possibility is to "correct" $K_{a\text{-}ICDI}$ to incorporate the effects of changes in Γ due to apoCaM binding. To do so we reason that for a mutant channel, $K_{a-CaM}^{MUT} = \lambda \cdot K_{a-CaM}$, where λ is a mutation specific constant and $K_{a\text{-}CaM}$ is the affinity of the wild-type channel for CaM (as above). We have previously quantified the effect of IQ domain on CaM binding by considering channels that lack the ICDI domain. Thus, Eq. 5 may be modified as:

$$\frac{CDI}{CDI_{\max}} = \frac{\lambda \cdot K_{a-CaM} \cdot [\mathrm{CaM}]_{\mathrm{free}} / [\mathrm{ICDI}]_{\mathrm{local}}}{K_{a-ICDI} + (1 + \lambda \cdot K_{a-CaM} \cdot [\mathrm{CaM}]_{\mathrm{free}}) / [\mathrm{ICDI}]_{\mathrm{local}}} \quad \text{-- (7)}$$

Once again, as the local concentration of the ICDI domain is high, $(1 + \lambda \cdot K_{a-CaM} \cdot [\mathrm{CaM}]_{\mathrm{free}})/[\mathrm{ICDI}]_{\mathrm{local}} \sim \lambda \cdot K_{a-CaM} \cdot [\mathrm{CaM}]_{\mathrm{free}}/[\mathrm{ICDI}]_{\mathrm{local}} = \lambda \cdot \Gamma$ As such Eq. 7 may be simplified as:

$$\frac{CDI}{CDI_{\max}} = \frac{\lambda \cdot \Gamma}{K_{a-ICDI} + \lambda \cdot \Gamma} = \frac{\Gamma}{K_{a-ICDI} / \lambda + \Gamma} \quad \text{-- (8)}$$

Since $\lambda = K_{a-CaM}^{MUT} / K_{a-CaM}$, Eq. 8 can be simplified as:

$$\frac{CDI}{CDI_{\max}} = \frac{\Gamma}{K_{a-ICDI}^{corr} + \Gamma} \quad \text{-- (9)}$$

Where $K_{a-ICDI}^{corr} = K_{a-ICDI}(K_{a-CaM} / K_{a-CaM}^{MUT})$. Of note, for mutations in the ICDI domain, λ=1. Thus, Eq 9 reduces to Eq. 6.

Supplementary References

1. Hulme, J. T., Yarov-Yarovoy, V., Lin, T. W., Scheuer, T., and Catterall, W. A. (2006) Autoinhibitory control of the CaV1.2 channel by its proteolytically processed distal C-terminal domain. *The Journal of physiology* **576**, 87-102
2. Hulme, J. T., Konoki, K., Lin, T. W., Gritsenko, M. A., Camp, D. G., Bigelow, D. J., and Catterall, W. A. (2005) Sites of proteolytic processing and noncovalent association of the distal C-terminal domain of CaV1.1 channels in skeletal muscle. *Proceedings of the National Academy of Sciences of the United States of America* **102**, 5274-5279
3. Sang, L., Dick, I. E., and Yue, D. T. (2016) Protein kinase A modulation of CaV1.4 calcium channels. *Nature communications* **7**, 12239
4. Bazzazi, H., Ben Johny, M., Adams, P. J., Soong, T. W., and Yue, D. T. (2013) Continuously tunable Ca(2+) regulation of RNA-edited CaV1.3 channels. *Cell reports* **5**, 367-377
5. Ben Johny, M., Yang, P. S., Bazzazi, H., and Yue, D. T. (2013) Dynamic switching of calmodulin interactions underlies Ca2+ regulation of CaV1.3 channels. *Nature communications* **4**, 1717
6. Liu, X., Yang, P. S., Yang, W., and Yue, D. T. (2010) Enzyme-inhibitor-like tuning of Ca(2+) channel connectivity with calmodulin. *Nature* **463**, 968-972
